# Global metabolomics revealed deviations from the metabolic aging clock in colorectal cancer patients

**DOI:** 10.7150/thno.87303

**Published:** 2024-02-04

**Authors:** Long Zhang, Shaobo Mo, Xiurui Zhu, C. James Chou, Bo Jin, Zhi Han, James Schilling, Weili Liao, Sheeno Thyparambil, Ruben Y. Luo, John C. Whitin, Lu Tian, Seema Nagpal, Scott R. Ceresnak, Harvey J. Cohen, Doff B. McElhinney, Karl G. Sylvester, Yangming Gong, Chen Fu, Xuefeng B. Ling, Junjie Peng

**Affiliations:** 1 Department of Colorectal Surgery, Fudan University Shanghai Cancer Center; Shanghai, China.; 2 Department of Oncology, Shanghai Medical College, Fudan University; Shanghai, China.; 3 Cancer Research Institute, Fudan University Shanghai Cancer Center; Shanghai, China.; 4 mProbe Inc.; Rockville, MD, USA.; 5 School of Medicine, Stanford University; Stanford, CA, USA.; 6 Shanghai Yunxiang Medical Technology Co., Ltd.; Shanghai, China.; 7 Tianjin Yunjian Medical Technology Co. Ltd.; Tianjin, China.; 8 Binhai Industrial Technology Research Institute, Zhejiang University; Tianjin, China.; 9 Shanghai Municipal Center for Disease Control and Prevention; Shanghai, China.; 10 Shanghai Clinical Research Center for Aging and Medicine; Shanghai, China.

**Keywords:** Aging, Global metabolomics, Metabolic aging clock, Colorectal advanced precancerous lesions, Colorectal cancer

## Abstract

**Background:** Markers of aging hold promise in the context of colorectal cancer (CRC) care. Utilizing high-resolution metabolomic profiling, we can unveil distinctive age-related patterns that have the potential to predict early CRC development. Our study aims to unearth a panel of aging markers and delve into the metabolomic alterations associated with aging and CRC.

**Methods:** We assembled a serum cohort comprising 5,649 individuals, consisting of 3,002 healthy volunteers, 715 patients diagnosed with colorectal advanced precancerous lesions (APL), and 1,932 CRC patients, to perform a comprehensive metabolomic analysis.

**Results:** We successfully identified unique age-associated patterns across 42 metabolic pathways. Moreover, we established a metabolic aging clock, comprising 9 key metabolites, using an elastic net regularized regression model that accurately estimates chronological age. Notably, we observed significant chronological disparities among the healthy population, APL patients, and CRC patients. By combining the analysis of circulative carcinoembryonic antigen levels with the categorization of individuals into the "hypo" metabolic aging subgroup, our blood test demonstrates the ability to detect APL and CRC with positive predictive values of 68.4% (64.3%, 72.2%) and 21.4% (17.8%, 25.9%), respectively.

**Conclusions:** This innovative approach utilizing our metabolic aging clock holds significant promise for accurately assessing biological age and enhancing our capacity to detect APL and CRC.

## Introduction

Aging is an inevitable life-long decline in physiological functions and is the major risk factor for high impact chronic diseases such as cancers and cardiovascular diseases 1. Aging involves extensive physiological changes and metabolic adaptations over decades 2. Modern “omics” platforms, including genomic, transcriptomic, proteomic, and metabolomic profiling assays, have provided new opportunities for the systematic and agnostic characterization of biological aging.

DNA methylation-based profiling, also known as “DNAm age”, is a powerful tool for predicting chronological age and assessing biological aging. It can be used across most tissues and cell types, and it incorporates composite clinical measures to capture risks for a wide range of age-related outcomes. Two of the most recent and promising DNAm age algorithms are “DNAm PhenoAge” 3 and “DNAm GrimAge” 4. DNAm PhenoAge was developed to predict multifactorial phenotypic age, while DNAm GrimAge was developed to study aging and age-related traits. Both algorithms have been shown to be strongly associated with mortality and other age-related health outcomes. DNAm age is a valuable tool for researchers and clinicians alike. It can be used to study the aging process, identify individuals at risk for age-related diseases, and develop personalized interventions to promote longevity.

Metabolic syndrome 5, a cluster of metabolic abnormalities, is age-related and regulated by key metabolic proteins such as mechanistic target of rapamycin (mTOR), AMP-activated protein kinase (AMPK), and insulin/insulin growth factor (IGF) 6, 7. Dysregulated metabolic control is a long-term cause of aging and increases the risk of chronic diseases. Metabolomic age models 8, 9, developed with unprecedented high-resolution metabolome coverage, assess biological age. Metabolomic 9 and epigenetic 10 aging clocks use different biomarkers, but both correlate with chronological age.

Age is the strongest risk factor for cancers, including advanced precancerous lesions (APL) and colorectal cancer (CRC). As people age, their risk of developing advanced polyps and CRC increases 11, 12. In this study, we used a deep metabolomic analysis of over 3,000 healthy individuals to investigate how the metabolome changes with age. We hypothesized that high-resolution metabolomic profiling could reveal unique age-associated patterns that could precisely predict chronological age. We also hypothesized that a metabolomic aging blood test could have clinical applications, such as assessing aging and detecting CRC early.

## Results

### Shanghai General Population and Cancer Center CRC Cohort Study

**Figure 1:**
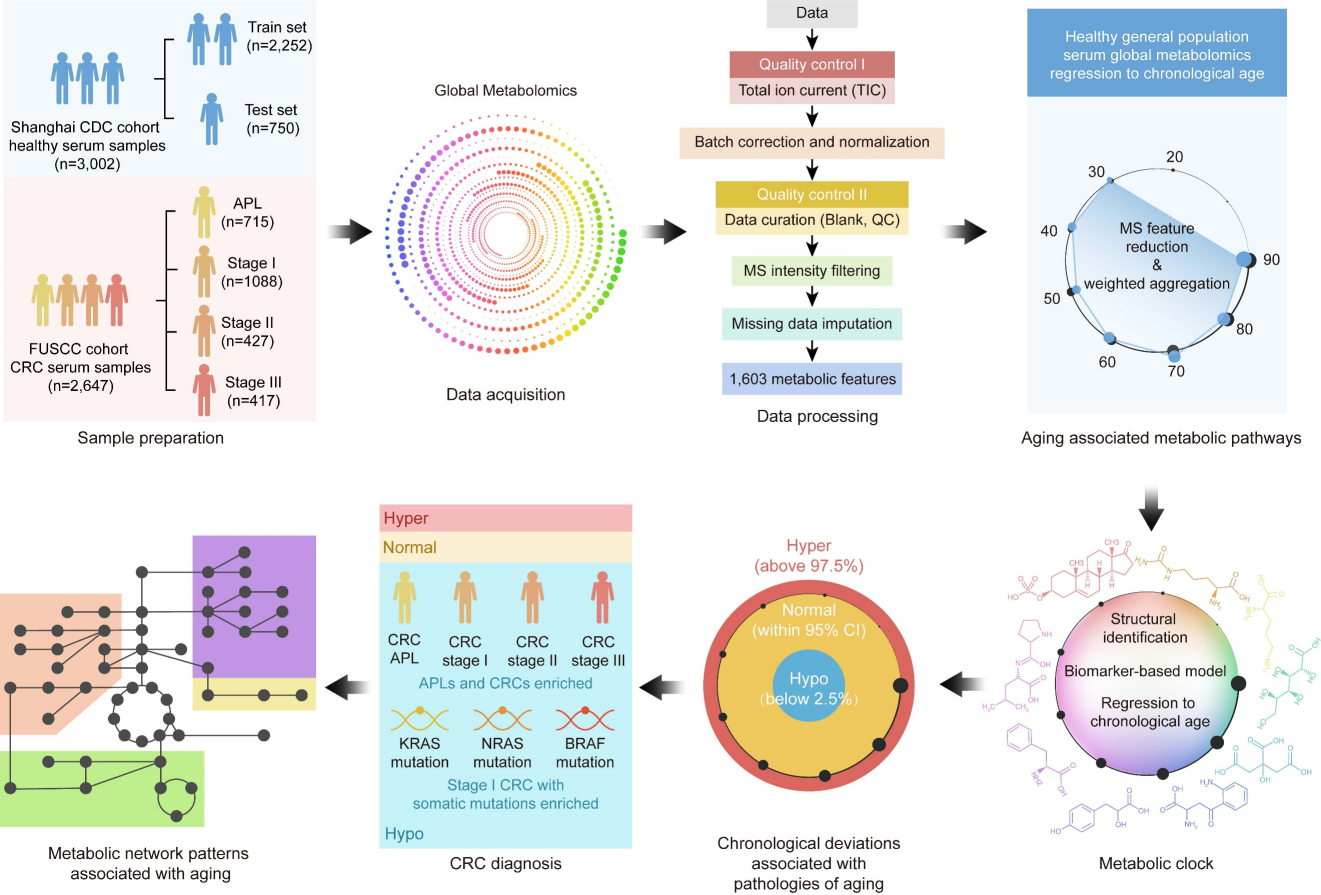
** Metabolomics data workflow.** In this study, 3,002 healthy volunteers and 2,647 patients with colorectal advanced precancerous lesions (APL) or colorectal cancer (CRC) were enrolled, and their serum samples were collected. High-resolution mass spectrometry was used for serum global metabolomics data acquisition, and 1,603 metabolic features were identified after data processing. After metabolic feature analysis, aging associated metabolic pathways were found to regress to chronological age. Further exploration of the metabolic features identified nine metabolites as the metabolic aging clock. Comparing actual chronological age and metabolic aging clock predicted age of healthy people using a 95% confidence interval (CI, 2.5%~97.5%) defined hyper, normal and hypo subgroups. Compared with healthy people, APL and CRC patients usually bearing somatic genetic mutations significantly fell into the hypo subgroup. At last, the underlying metabolic patterns associated with aging were revealed.

The study design and methods are outlined in Figure 1. We collected pretreatment serum samples (Supplementary Material 1) from 3,002 healthy individuals from the Shanghai Centers for Disease Control and Prevention (CDC), 715 patients with advanced precancerous lesions (APL), and 1,932 CRC patients without known contribution from germline causes or significant family history of cancer or inflammatory bowel disease (i.e., patients whose CRC is not thought to be caused by a genetic mutation or a strong family history of cancer). Demographic data are summarized in Table 1.

### Unique Metabolomic Patterns Predict Chronological Age

Using high-resolution mass spectrometry (Supplementary Material 2) to profile blood metabolomes, we identified 1,603 metabolic features. Of these, 157 were associated with aging (Pearson correlation, 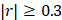). We aggregated the aging associated features into KEGG pathways and calculated the value of each pathway as the weighted sum of the normalized measurement values of aging associated metabolites on the pathway divided by the number of mapped metabolites (Supplementary Material 3). Using an elastic net approach, we regressed these 59 pathways on chronological age and found that 42 of them contributed to the multivariate regression with positive importance scores.

**Figure 2:**
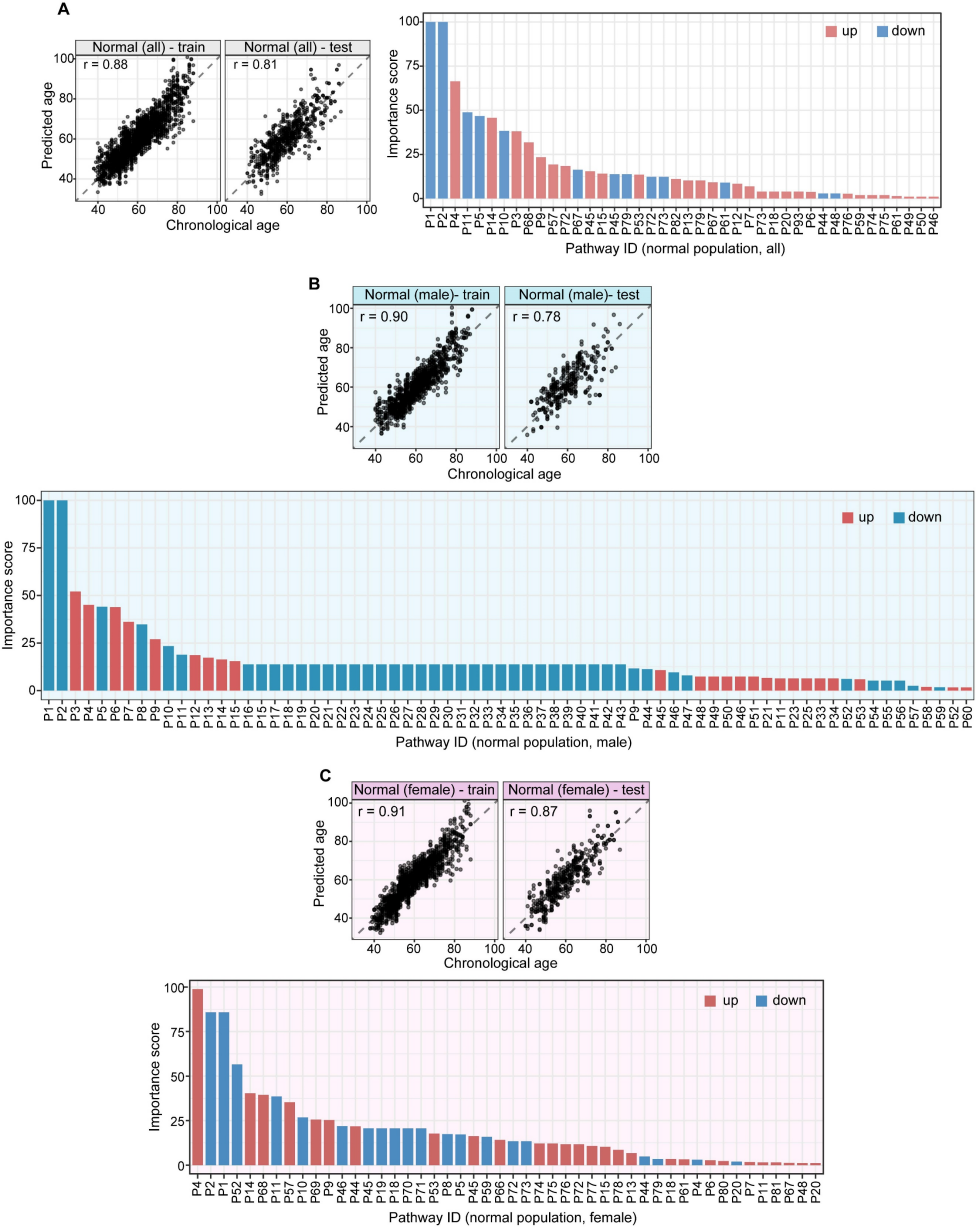
** A pathway based metabolic aging clock from the general population.** Importance scores of underlying pathways from untargeted metabolic profiling (training set:75%; testing set:25%). **(A).** Prediction of metabolic ages from all normal population (both sexes). **(B).** Prediction of metabolic ages from male normal population. **(C).** Prediction of metabolic ages from female normal population. The pathway IDs in the figure are as follows: P1, Steroid hormone biosynthesis; P2, Bile secretion; P3, Phenylalanine metabolism; P4, ABC transporters; P5, Metabolism of xenobiotics by cytochrome P450; P6, 2-Oxocarboxylic acid metabolism; P7, Lysine degradation; P8, Biosynthesis of amino acids; P9, Pyrimidine metabolism; P10, Chemical carcinogenesis; P11, Histidine metabolism; P12, Amino sugar and nucleotide sugar metabolism; P13, Protein digestion and absorption; P14, Riboflavin metabolism; P15, Pentose and glucuronate interconversions; P16, Arginine biosynthesis; P17, Ascorbate and aldarate metabolism; P18, Glyoxylate and dicarboxylate metabolism; P19, Carbon metabolism; P20, Taste transduction; P21, Ferroptosis; P22, Proximal tubule bicarbonate reclamation; P23, D-Glutamine and D-glutamate metabolism; P24, Neomycin, kanamycin and gentamicin biosynthesis; P25, Nitrogen metabolism; P26, FoxO signaling pathway; P27, Phospholipase D signaling pathway; P28, Gap junction; P29, Circadian entrainment; P30, Long-term potentiation; P31, Synaptic vesicle cycle; P32, Retrograde endocannabinoid signaling; P33, Glutamatergic synapse; P34, GABAergic synapse; P35, Long-term depression; P36, Amyotrophic lateral sclerosis; P37, Huntington disease; P38, Spinocerebellar ataxia; P39, Cocaine addiction; P40, Amphetamine addiction; P41, Nicotine addiction; P42, Alcoholism; P43, Sulfur relay system; P44, Valine, leucine and isoleucine degradation; P45, Glutathione metabolism; P46, Butanoate metabolism; P47, Antifolate resistance; P48, Valine, leucine and isoleucine biosynthesis; P49, Fatty acid degradation; P50, Vitamin B6 metabolism; P51, Pantothenate and CoA biosynthesis; P52, Porphyrin and chlorophyll metabolism; P53, Adrenergic signaling in cardiomyocytes; P54, Taurine and hypotaurine metabolism; P55, Sulfur metabolism; P56, Neuroactive ligand-receptor interaction; P57, Ubiquinone and other terpenoid-quinone biosynthesis; P58, Pentose phosphate pathway; P59, Tryptophan metabolism; P60, beta-Alanine metabolism; P61, Arginine and proline metabolism; P62, Central carbon metabolism in cancer; P63, Aminoacyl-tRNA biosynthesis; P64, Mineral absorption; P65, Glycolysis / Gluconeogenesis; P66, Pyruvate metabolism; P67, Glycine, serine and threonine metabolism; P68, Citrate cycle (TCA cycle); P69, Insulin resistance; P70, Sphingolipid metabolism; P71, Sphingolipid signaling pathway; P72, Nicotinate and nicotinamide metabolism; P73, Alanine, aspartate and glutamate metabolism; P74, Serotonergic synapse; P75, Drug metabolism - cytochrome P450; P76, D-Arginine and D-ornithine metabolism; P77, Purine metabolism; P78, Biotin metabolism; P79, Cysteine and methionine metabolism; P80, Phenylalanine, tyrosine and tryptophan biosynthesis; P81, Steroid biosynthesis; P82, Dopaminergic synapse; P83, Galactose metabolism; P84, Fructose and mannose metabolism; P85, Caffeine metabolism; P86, Drug metabolism - other enzymes; P87, Phosphonate and phosphinate metabolism; P88, Glycosaminoglycan biosynthesis - heparan sulfate / heparin; P89, Cholinergic synapse; P90, Folate biosynthesis; P91, Propanoate metabolism; P92, Primary bile acid biosynthesis; P93, Glucagon signaling pathway; P94, Tyrosine metabolism.

The 42 pathways collectively achieved improved regression on chronological age (Figure 2A, Supplementary Figure 1): training, 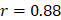, 95%CI 0.88-0.89, 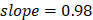, 95%CI 0.95-1.00; testing, 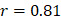, 95%CI 0.80-0.83, 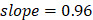, 95%CI 0.90-1.01. The top 10 metabolic pathways, ranked by Pearson correlation to chronological age, were steroid hormone biosynthesis, bile secretion, ABC transporters, histidine metabolism, metabolism of xenobiotics by cytochrome P450, riboflavin metabolism, chemical carcinogenesis, phenylalanine metabolism, citrate cycle, and pyrimidine metabolism.

We performed the pathway-based multivariate regression analysis with men, and women separately. Using the same statistical pipeline for the general population (Figure 2A, 42 pathways), we identified 70 and 48 metabolic pathways (Figure 2B/2C) for the multivariate analyses of men and women populations, respectively. The Pearson correlation coefficients were 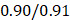 (men/women) in training and 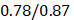
(men/women) in testing respectively (Figure 2B/2C). The Pearson correlation coefficients among these three populations, all/men/women were statistically significant (p-value = 0.0001). The rankings of the importance scores of the significant aging-correlating pathway features were similar among all, men, and women (Figure 2A/2B/2C). For example, the steroid hormone biosynthesis pathway ranked top 1 in both the all's and men's models and ranked top 3 in the women's model.

### Nine-Metabolite Metabolic Aging Clock

**Figure 3:**
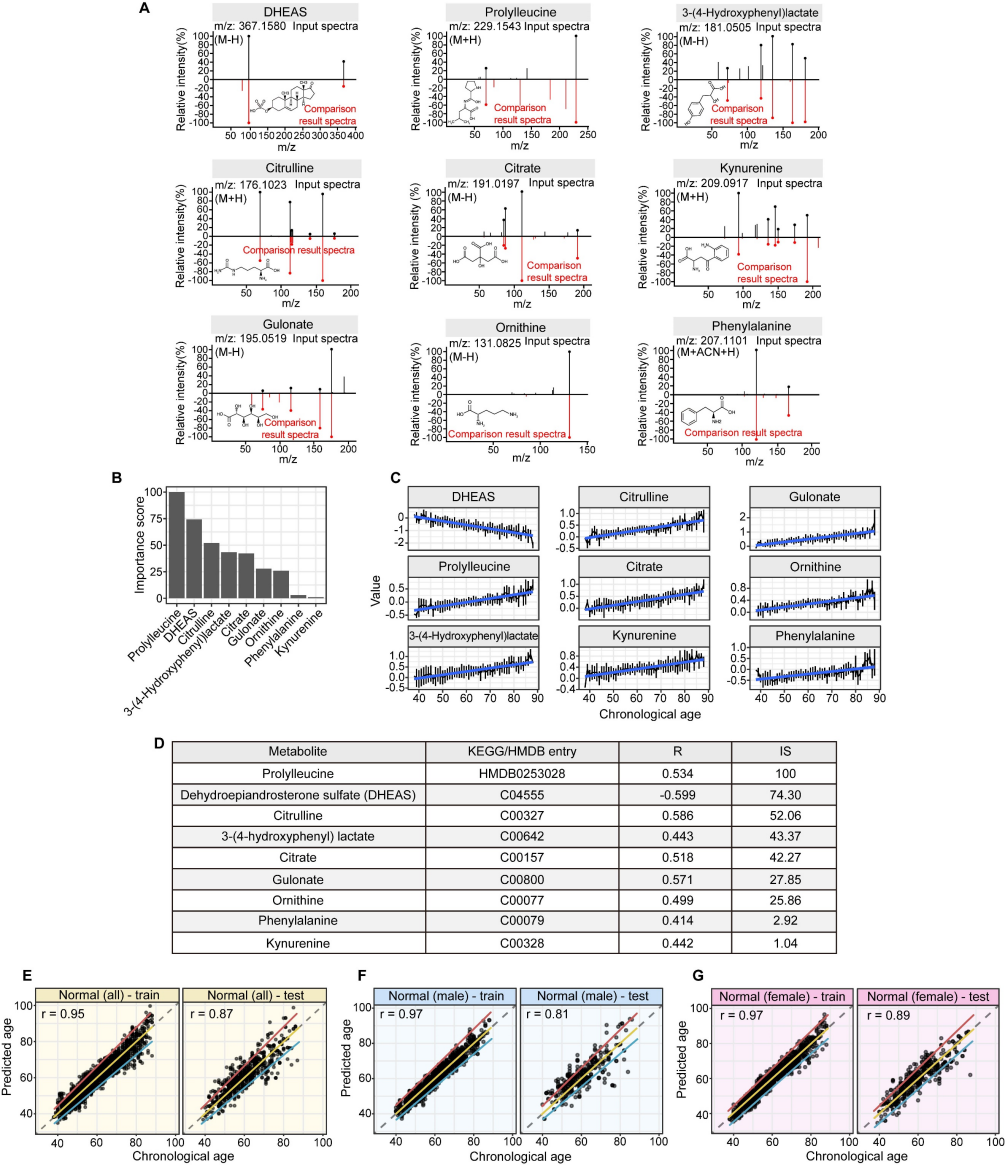
** Structural identification of aging metabolite biomarkers and nine-metabolite-based metabolic aging clock. (A).** Confirmation of the metabolites predicting chronological age by standard compounds. **(B).** Importance scores of the identified biomarkers in a biomarker-based metabolic aging clock. **(C).** Univariate trajectories of aging biomarkers as a function of the chronological age.** (D).** Nine compound biomarkers ranked by the importance scores in the aging clock model. KEGG: Kyoto Encyclopedia of Genes and Genomes; HMDB; Human Metabolome Database; R: Pearson Correlation Coefficient for regressing on the chronological age; IS: importance score in the multivariate model. **(E-G).** Prediction of metabolic ages in the training (75%) and testing (25%) sets from all the healthy general population (both sexes), from the male healthy population, and from the female healthy population.

Linear modeling links metabolic pathways to aging. We selected from the significant aging-associated metabolic features to identify a panel of metabolite biomarkers, called the "metabolic aging clock," to assess aging. The nine metabolites in the metabolic aging clock were identified (Supplementary Material 4) using a combination of level 1 compound identification and multivariate regression (Supplementary Figure 3). The nine metabolites are shown in Figure 3A. The results of both the multivariate analysis (importance scores, IS, Figure 3B) and univariate analysis (Pearson correlation coefficient, r, Figure 3C) are summarized in Figure 3D. The metabolic aging clock achieved improved regression on chronological age, with a Pearson correlation coefficient 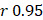 (95%CI 0.946-0.954) and 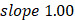 (95%CI 0.97-1.03) in the training, and 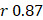, 95%CI 0.85-0.89, 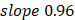, 95%CI 0.90-1.03 in the testing set (Figure 3E). The results were similar for all subjects, men, and women (Figure 3E-G): the Pearson correlation coefficient (r) 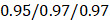 (all/men/women, *p*-value, 0.24) in training and 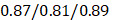
(all/men/women, *p*-value < 0.0001) in testing respectively.

### Hypo-aging phenotypes in CRC patients

We used a metabolic clock to assess the metabolic ages of healthy people. We defined the "Δ metabolic aging" as the difference between the predicted and actual chronological ages. We used a 95% confidence interval (CI, 2.5%~97.5%) to define the "normal" (within the 95% CI), "hyper" (above the 97.5%), and "hypo" (below the 2.5%) Δ metabolic aging subgroup membership.

**Figure 4:**
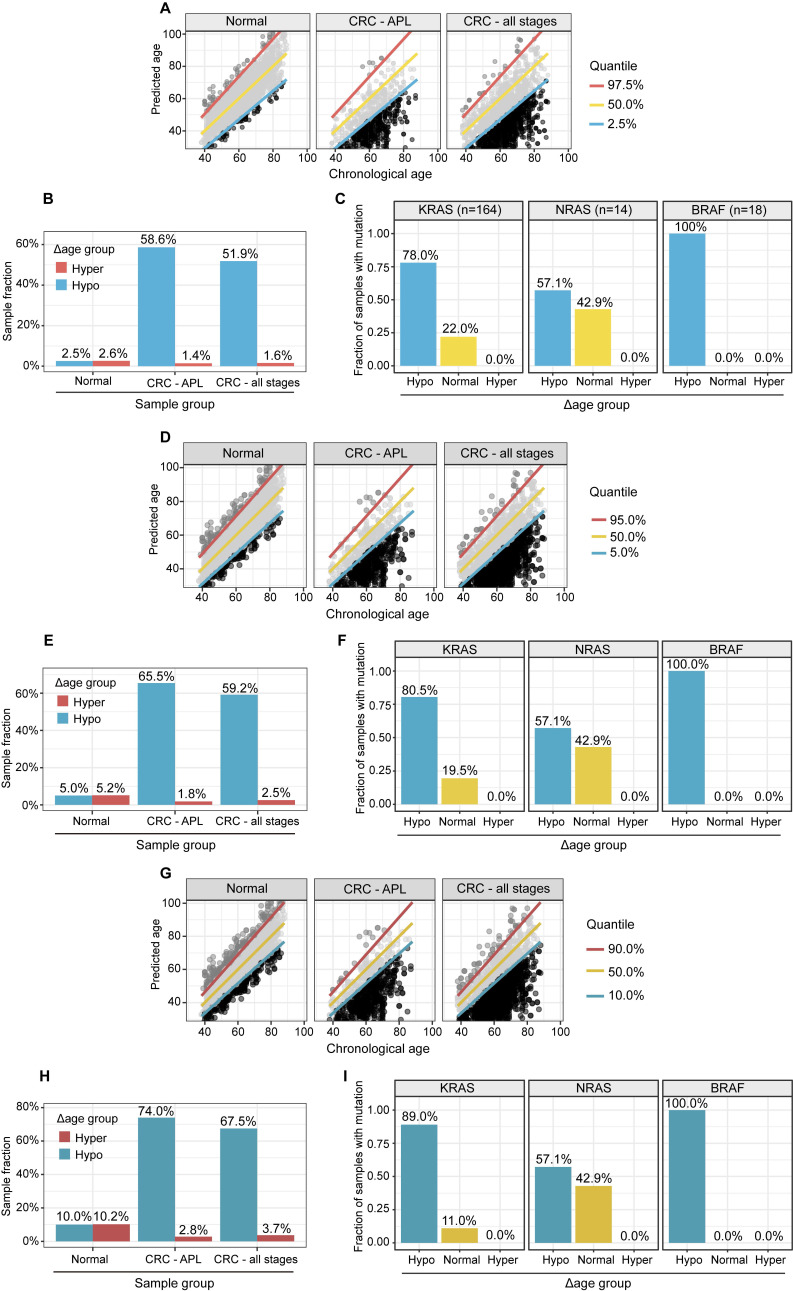
** APL and CRC (all stages) samples have significantly higher fractions, and stage I CRC patients with gene mutations are more likely to have “hypo” metabolic age. (A, D, G).** XY plot of the prediction of metabolic ages as a function of the chronological age in the normal general and CRC populations. (**B, E, H).** Sample fractions of the total in the hypo or hyper metabolic age subgroups. **(C, F, I).** KRAS, NRAS and BRAF mutations were significantly enriched in the hypo Δ age group (p < 0.01). The percentages were fractions of stage I CRC samples with corresponding mutations.** (A-C):** Quantile 2.5%, 50%, 97.5%. **(D-F):** Quantile 5%, 50%, 95%.** (G-I):** Quantile 10%, 50%, 90%.

Compared to healthy people, individuals with "hypo" membership (Figure 4A) were more likely to have APL or CRC, regardless of the specific CI thresholds used to define the "normal", "hyper", and "hypo" Δ metabolic aging subgroups (Figure 4B, 4D-E, 4G-H). This suggests that a "hypo" metabolic aging phenotype is associated with an increased risk of APL and CRC (Supplementary Material 5, Supplementary Table 1, 2, and 3).

### Hypo-Aging Phenotypes and Their Potential Clinical Utility for Improving Colorectal Cancer Diagnosis

The prevalence of APL and CRC in the general population is 7.6% and 0.7% 13, respectively. Because there were many more APLs and CRCs in the hypo Δ metabolic aging subgroup, we investigated whether the metabolic aging clock could be used to detect CRC.

Specifically, we used hypo Δ metabolic aging subgroup membership to diagnose CRC status. After adjusting for the true incidence rate of CRC in the general population, the positive predictive values (PPVs, Table 2, Supplementary Table 2C) for APL and all CRC stages were 65.5% (62.3-68.5%) and 12.7% (10.0-15.9%), respectively.

Although serum carcinoembryonic antigen (CEA) does not have sufficient sensitivity or specificity to diagnose CRC (Table 2 PPV: APL, 5.2% (4.6-5.7%); CRC, 0.4% (0.3-0.7%)), it is still considered the most important biomarker for detecting CRC.

By removing samples with normal CEA measurements from the positives predicted by the metabolic clock classifier, we created a multi-target panel (nine metabolites plus CEA) that improved the PPVs to 68.4% ((64.3%, 72.2%), p=1.3x10^-5^) for APL, and 21.4% (17.8%-25.9%), p= 1.2x10^-10^) for CRC. This suggests that CEA and the metabolic clock work together to improve APL/CRC diagnosis.

### Metabolic Aging and CRC Mutation Profiles

**Figure 5:**
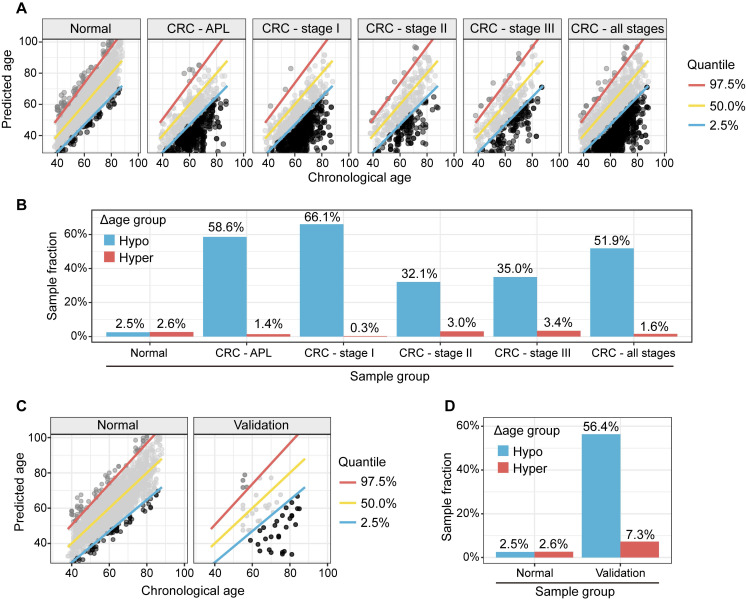
** Metabolic clock analysis of APL and stage I CRC subjects. (A).** XY plot of the predicted metabolic ages as a function of chronological ages. Samples below the 2.5% quantile line were defined as hypo Δ metabolic age subgroup and those above the 97.5% one as hyper Δ metabolic age subgroup. **(B).** Enrichment of APL and CRC stage I subjects in Δ metabolic age “hypo” subgroup. **(C).** XY plot of the prediction of metabolic ages as a function of the chronological age in the normal general and CRC populations. **(D).** Sample fractions of the total in the hypo or hyper Δ metabolic age subgroups.

To study the genomic mutation patterns of colorectal cancers (CRCs) in the metabolic Δ aging subgroups, we profiled 412 samples from patients with stage I CRC (Supplementary Table 3A). Among the 164/412 (39.8%) patients with KRAS, 14/412 (3.4%) with NRAS, and 18/412 (4.4%) with BRAF mutations/stage I CRCs (Supplementary Table 3B), 78.0% (71.3%, 84.2%) KRAS, 57.1% (28.8%, 85.7%) NRAS, and 100.0% (100.0%, 100.0%) BRAF mutants were found to be in the "hypo" metabolic age group (Figure 4C, 4F, and 4I). For reference, 66.1% of stage I CRC patients fell into the "hypo" subgroup (Figure 5).

## Discussion

In this study, we used high-resolution mass spectrometry to identify key metabolic changes that correlate with chronological age in a healthy general population. We also developed a metabolic aging clock, a predictive model based on nine blood metabolites, to depict the age clock in the general population and in patients with colorectal cancer.

Previous studies have shown that certain biomarkers in our metabolic aging clock are associated with the aging process. Levels of kynurenine and phenylalanine increase with age, while levels of dehydroepiandrosterone (DHEAS) sulfate decrease. Kynurenine is produced by indoleamine 2,3-dioxygenase and tryptophan 2,3-dioxygenase from tryptophan. High levels of circulating kynurenine are thought to be a primary driver of aging 14-18, linked to increased frailty and mortality in humans. DHEAS peaks around age 20 and then gradually declines over time. By age 70, DHEA-S levels are about 20-30% lower than in younger adults 19-22. Recently, researchers have reported that circulating phenylalanine also increases with age and is closely related to heart aging 23. Older people also have a slower plasma clearance rate of phenylalanine, resulting in age-dependent increases 24. Metabolites such as citrulline and ornithine are involved in the L-arginine/nitric oxide pathway and are thought to have anti-aging effects. The upregulation of circulating citrulline and ornithine could be a homeostatic response to the aging vesicular system in healthy individuals. Citrate, 3-(4-hdyroxyphenyl) lactate, and gulonate are closely related to cellular energy metabolism.

An accumulation of these metabolites could indicate a shift in cellular metabolism between either glycolysis or mitochondrial respiration. Prolylleucine is associated with muscle tone, type 2 diabetes, and insulin resistance, conditions that are highly associated with age 25, 26. Prolylleucine levels are upregulated in males with insulin resistance and are significantly upregulated in people with type 2 diabetes, regardless of sex 25. Prolylleucine has been proposed as a biomarker for type 2 diabetes 25.

Our findings that patients with CRC have a hypo metabolic age are consistent with a recent study of the PhenoAge clock (CpG markers: n=513), which showed a similar hypo-aging trend among high-risk CRC patients 27. To develop a binary classifier for CRC assessment, we applied a random forest method to nine metabolite aging biomarkers. This improved the performance of the metabolic aging clock predictor of CRC status modestly: APL, 65.5% (62.3%, 68.5%); CRC, 12.7% (10.0%, 15.9%) (Table 2). The nine metabolic aging clock markers were originally discovered to regress to chronological age, but they can also be used directly with a more classical approach (a random forest method) to train a cancer binary classifier. This provides direct evidence to support the application of the metabolic aging clock in cancer assessment.

In this study, we validated our hypothesis that the metabolic aging clock and its hypo-aging membership could improve the early diagnosis of colorectal cancer (CRC). Our metabolic clock panel results significantly outperformed previous findings, with a much-improved positive predictive value (PPV) for APL (65.5%) and CRC (12.7%) (Table 2) than Cologuard^TM^ 18 (20.0% for APL and 3.72% for CRC) and Septin 9 methylation tests (9.5% for APL and 2.3% for CRC) 28. We further demonstrated that the CRC marker CEA could work together with our aging clock to improve the PPVs to identify APL (68.4%) and all stages of CRC cases (21.4%) (Table 2). Therefore, our models achieved higher PPV values to identify APL and stage I CRC subjects than current clinically available diagnostic methods, using either the metabolic aging clock panel or the panel plus CEA. A good disease marker usually becomes more relevant with the severity of the disease, as it should accurately measure the presence and progression of the disease. However, our predictive performance with precancerous lesions and different CRC stages is counterintuitive. We hypothesize that this may be due to the different mechanisms of action between tumor genesis and later tumor progression. Tumor genesis is the process by which a normal cell transforms into a cancer cell, while tumor progression is the process by which cancer cells grow and spread throughout the body. Future research is needed to address this.

Clinically, KRAS and BRAF mutations are associated with a poor prognosis 29, 30. Patients with CRC and BRAF mutations do not respond to cetuximab, and all but one patient with any of the three mutations did not respond 31. Patients with any of the three mutations had a poor response rate (7.1%) and reduced survival (progression-free survival = 8 months) compared with wild-type counterparts (74.4% and 11.6 months). Our study showed that hypo-aging individuals were also highly enriched in the stage I CRC patient population with KRAS and BRAF mutations. A hypo-aging phenotype is typically associated with a less differentiated CRC phenotype and is usually less responsive to chemotherapeutic agents 32. Epigenetic data suggests that decelerated epigenetic aging is associated with a poorer prognosis and lower overall survival rate in CRC 33. Our study bridges the gap between clinical observations and epigenetic studies of CRC patients with KRAS and BRAF mutations in stage I CRC through a metabolic aging clock, illustrating a spectrum of malignancy with metabolic aging deviations in this stage.

Our study has several potential limitations:1. Enrolled patients were not required to take a germline mutation test, so it is possible that a small number of patients without a family history of CRC had germline mutations; 2. Our metabolic clock analytics for early CRC detection may have been confounded by pre-analytic variables and cohort differences in sex and age, which differed between the general population and CRC cohorts. A stronger single-site study design would help rule out the possibility of systematic confounding related to differences in blood collection and processing. In a subset of our cohort enrolled at Shanghai CDC, identical blood collection was performed in healthy controls and CRC patients (n=55) who were identified as part of the CDC screening. Similar hypo-aging membership patterns were observed in this subgroup of CRC patients (Figure 5C-5D), which supports the validity of our CRC results (Figure 4A-4B) from Fudan University Shanghai Cancer Center. In addition, we built 100 random models (Supplementary Material 6) using the statistical pipeline already established and compare if the deviation of the CRC/APL cohort from the CI derived from the general population is immanent/systematic (=bias) or specific for the age-related signature. Supplementary Figure 4A/B showed that our results are biologically meaningful and statistically significant. Our findings are unlikely to be due to technical bias; 3. This study is not designed to test the hypothesis that KRAS, NRAS, and BRAF mutations in other tumors cause hypometabolic age, or vice versa. Future studies with multi-cancer detection cohorts that include both pretreatment liquid and tissue biopsy samples could test these hypotheses, but they are beyond the scope of this study and is a limitation of the current study. 4. This study cannot test whether any metabolic age biomarkers change specifically in colorectal cancer (CRC). Although we plan to explore the metabolic aging clock's clinical utility in other high-impact diseases, including other cancers, the generalizability of this approach needs to be validated with additional independent cohorts that can demonstrate minimal false positives and localization, evaluate the implementation and real-world performance of the test in clinical practice, confirm the results in a population with no known diagnosis, and validate the clinical utility in a high-risk population.

## Conclusions

**Figure 6:**
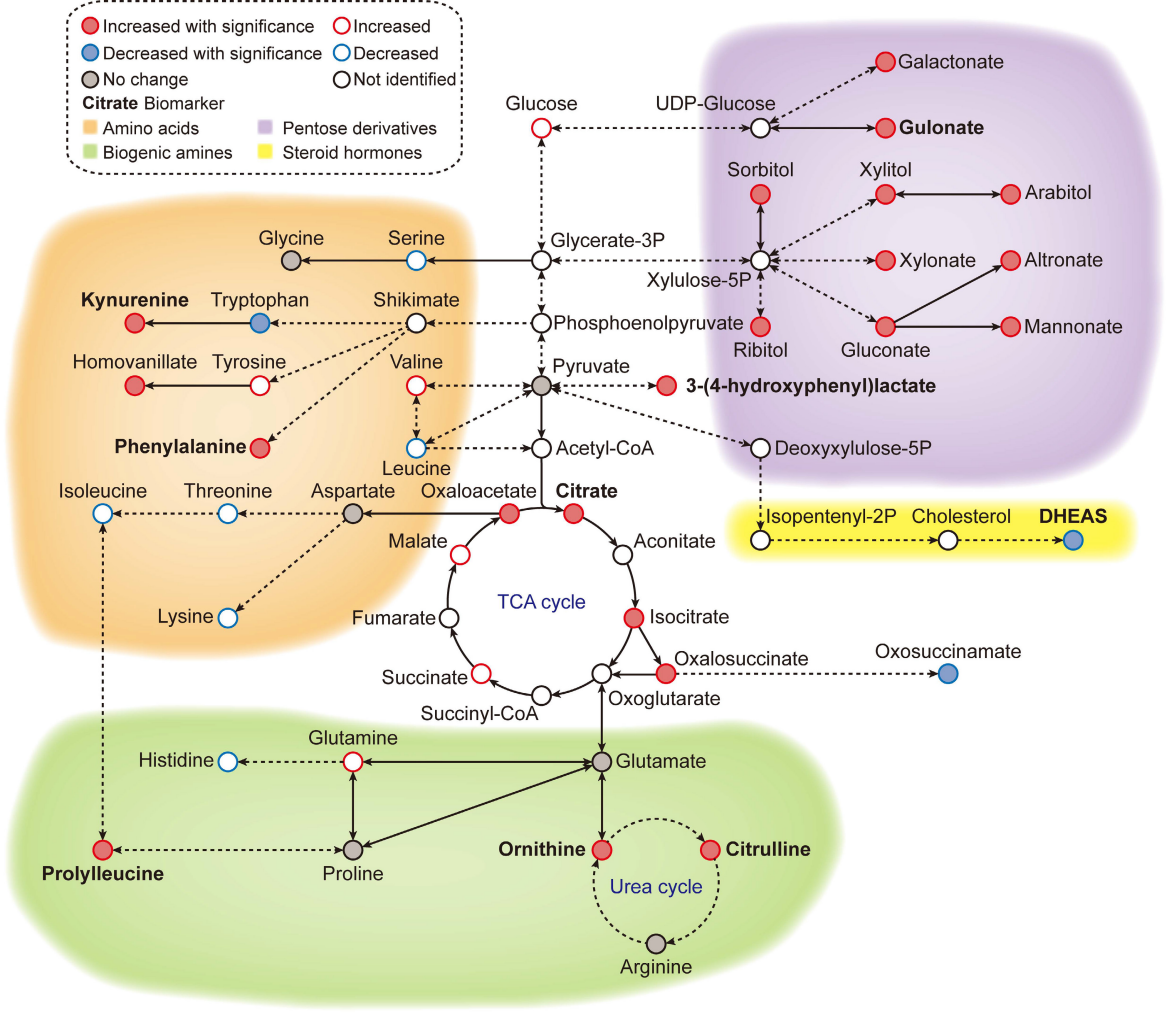
**Metabolic overview of the reference ageotype.** With global metabolomics, many metabolites were quantified and identified, forming a metabolic network of 4 clusters: amino acid metabolism (orange), biogenic amine metabolism (green), pentose/glucuronate conversions (purple) and steroid hormone biosynthesis (yellow). The names of the discovered biomarkers were highlighted in bold. Correlation between metabolites and aging was visualized with colored edges and fills in the nodes.

Our global metabolomic analysis revealed high-resolution metabolomic pattern changes (Figure 6) associated with aging progression and colorectal cancer (CRC) status. Our findings could lead to new approaches to longevity medicine and early detection of CRC, but further validation is needed in large, blinded clinical trials.

## Methods

### Study design and ethical approval

This study (Figure 1) was approved by the Shanghai Municipal Center for Disease Control and Prevention Ethical Review Committee (No. 2019-4) and the Ethical Committee and Institutional Review Board of Fudan University Shanghai Cancer Center (No. 1902197-15).

### Healthy general population subjects in the Shanghai CDC cohort

To be eligible for the study, participants had to be at least 18 years old, not taking any medication for high-impact chronic diseases (such as cardiovascular diseases or diabetes mellitus) and have no history of tumors or cancers. Participants were followed for three years to identify any new cancer lesions or chronic diseases, and those who developed these conditions were excluded from the study.

### Sporadic colorectal cancer (CRC) patients in Fudan University Shanghai Cancer Center cohort

We excluded patients with Lynch syndrome and FAP, which account for 5-7% of all colorectal cases and are mainly characterized by early-onset colorectal cancer and multiple polyps. These conditions may result in unique aging features. Our CRC subjects were defined as those with cancers that arise from the colorectum without known contribution from germline causes, a significant family history of cancer, or inflammatory bowel disease. We constructed the cohort by screening cases to exclude patients with common familial colorectal cancer according to their family history and clinical profile. For example, we excluded patients with Lynch syndrome according to the Amsterdam II criteria. Thus, our study focused on the aging characteristics of sporadic colorectal cancer. We also excluded patients who were taking any medication for high-impact chronic diseases (such as cardiovascular diseases or diabetes mellitus). The collected samples were derived from the Department of Biobank, Fudan University Shanghai Cancer Center.

### Sample preparation

Our cohort sera were collected from cancer patients before chemotherapy or radiotherapy administration. We used 3,002 serum samples (Supplementary Material 1) from healthy general population subjects in the Shanghai CDC cohort, 715 serum samples from patients with advanced precancerous lesions (APL), and 1,932 serum samples from CRC patients from Fudan University Shanghai Cancer Center in this study, after excluding ineligible participants. Demographic data are summarized in Table 1. We collected whole blood samples from patients and generated sera following the standard operating procedure (SOP) described in Supplementary Material 1 34.

### MS acquisition, QA/QC, annotation, structural identification

The MS analytic pipeline for data acquisition, QA/QC, annotation, and structure identification was described in detail in Supplementary Material 2 35-61.

### Identification of age associated metabolic pathways

We were among the first groups to propose a pathway-based computational methodology for chronological event prediction with global metabolomics 62. Detailed analyzing method was described in Supplementary Material 3 47-65. We provide a Supplementary Material 7 (Supplementary Table 5) describing aging associated KEGG metabolic pathways and their associated mapped metabolomic features.

### Construction of a metabolic aging clock with nine compounds

Through an elastic net regularized regression (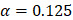, and 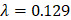), a metabolic aging clock was trained with the metabolite biomarker candidates. Detailed analyzing method was described in Supplementary Material 4 42, 43. Evidence to support the appropriateness to use of ElasticNet is described in Supplementary Material 8 and Supplementary Figure 5 in this study.

### Metabolic aging clock for CRC diagnosis

To leverage the clinical utility for the chronological deviations observed in CRC subjects, our metabolic panel classifies all samples in the hypo-aging group as CRC. In addition, a multi-target panel was assembled by removing samples with normal carcinoembryonic antigen (CEA) measurements (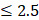 μg/L for non-smokers and 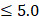 μg/L for smokers) from the positives assigned by the metabolic clock, since CEA is a known CRC biomarker 66. To simulate CRC incidence in general population, predictions from the testing dataset and the CRC cohort were bootstrapped with replacement at 30x coverage to an incidence of 760/10,023 for CRC APL samples and 65/10,023 for all stages of CRC samples 13. Positive predictive values (PPVs) were calculated with the ratio of true positive counts to total predictive positive counts. PPV CIs were calculated with logit transformation and central limit theorem assumption as previously published 67.

### Metabolic clock in CRC mutation status

A subpopulation of 412 subjects in CRC stage I group were profiled with *KRAS*, *NRAS* and *BRAF* mutations in the tumor tissue samples. All mutants were assigned into hypo-, normal and hyper-aging groups by the metabolite-based metabolic aging clock. Then, sample fractions of mutant samples in each group were calculated for each mutation to reveal enrichment trending. Finally, 95% CIs of sample fractions were calculated by bootstrapping the classification results of mutant samples with replacement at the same size for 10,000 times to derive the 2.5% and 97.5% quantiles.

### Aging metabolic network construction

To visualize the metabolic network underlying the global metabolomic aging patterns, age correlating metabolites were annotated to various metabolic modules and pathways.

## Supplementary Material

Supplementary methods, figures and tables.

## Figures and Tables

**Table 1:** Demographics showing sample distributions across age and sex in normal and CRC populations.

	Normal	APL	CRC - stage I	CRC - stage II	CRC - stage III
**Total**	3002	715	1088	427	417
**Age group, N (%)**					
**(30,40]**	21 (0.7)	14 (2)	12 (1.1)	9 (2.1)	17 (4.1)
**(40,50]**	503 (16.8)	105 (14.7)	148 (13.6)	55 (12.9)	60 (14.4)
**(50,60]**	1124 (37.4)	219 (30.6)	295 (27.1)	104 (24.4)	123 (29.5)
**(60,70]**	851 (28.3)	268 (37.5)	426 (39.2)	161 (37.7)	138 (33.1)
**(70,80]**	387 (12.9)	90 (12.6)	175 (16.1)	80 (18.7)	66 (15.8)
**(80,90]**	116 (3.9)	19 (2.7)	32 (2.9)	18 (4.2)	13 (3.1)
**Sex, N (%)**					
**Male**	1312 (43.7)	410 (57.3)	623 (57.3)	272 (63.7)	234 (56.1)
**Female**	1690 (56.3)	305 (42.7)	465 (42.7)	155 (36.3)	183 (43.9)

**Table 2:** Comparison of positive predictive values (PPVs) of tests for CRC diagnosis.

	APL	CRC - all stages
**Metabolic clock panel ^a^**	65.5% (62.3%, 68.5%)	12.7% (10.0%, 15.9%)
**Multi-target panel ^b^**	68.4% (64.3%, 72.2%)	21.4% (17.8%, 25.9%)
**CEA**	5.2% (4.6%, 5.7%)	0.4% (0.3%, 0.7%)
**Cologuard ^c^**	20.0% (18.0%, 22.0%)	3.72% (2.85%, 4.76%)
**Septin 9 methylation ^d^**	9.5% (9.1%, 9.9%)	2.3% (1.8%. 2.9%)

a. Contains the 9 metabolic biomarkers. b. Contains the 9 metabolic biomarkers and carcinoembryonic antigen (CEA). c. Data cited from reference 68. d. Data cited from external source reference 28.
