# Supplementary material for: Global metabolomics revealed deviations from the metabolic aging clock in colorectal cancer patients
Source: Theranostics. 2024 Feb 4;14(4):1602–14. doi: 10.7150/thno.87303 (PMC10879879; doi:10.7150/thno.87303)
Supplement: Supplementary file 1 — Supplementary methods, figures and tables. [file thnov14p1602s1.pdf]

Supplementary Material 1

Standard Operating Procedure:

Serum sample collection and processing for molecular analysis

Serum is the liquid fraction of whole blood that is collected after the blood is allowed to clot. The clot is removed by centrifugation and the resulting supernatant, designated serum, is carefully removed using a Pasteur pipette.

INSTRUCTIONS for Sample Collection:

1. **BLOOD**: Collect **blood** via venipuncture into vacutainers (see below):

BLOOD SAMPLE COLLECTION AND PROCESSING[1]:

Serum:

1. Using sterile technique, perform venipuncture and collect 2-mL blood into a vacutainer containing no pro- or anticoagulant (red top or tiger-top tube). Document date and time of collection.
2. Whole blood can be stored at 4-8°C within 12 hours before the serum is separated, but it must not be frozen.
3. Allow blood to clot by leaving tube undisturbed at room temperature for 30 min*.
4. Centrifuge tubes at 1000 × g for 10 min in a refrigerated centrifuge. Document date and time. The resulting supernatant is designated serum.

5. Following centrifugation, it is important to immediately transfer the liquid component (serum) into a clean freezer compatible polypropylene tube using a Pasteur pipette. The samples should be maintained at 2-8°C while handling.

6. The serum should be apportioned into 2 × 250 μL aliquots, and labeled as: specimen type: blood (serum).

7. Freeze immediately at -20°C within 6 hours from the time of collection, and transfer to -80°C within 7 days for long-term storage. Document date and time。

8. Record the following information in the CASE REPORT FORM:

Specimen matrix

Collection date and time

Centrifugation times

Date and time placed in storage

Freezer ID# and temperature

Initials

It is important to avoid freeze-thaw cycles because this is detrimental to many serum and plasma components. Samples that are hemolyzed, icteric or lipemic can invalidate certain tests, and therefore should be documented.

[1] J Proteome Res. 2009 Jan; 8(1): 113–117. (PMID: 19072545)

*in order to obtain serum of high quality, blood samples should be allowed time to form a clot at room temperature for 30-60 min.

Serum tubes

For more information on the tubes, please visit Becton-Dickinson Resources:

https://www.bd.com/resource.aspx?IDX=7220

Red	No anticoagulant
Lavender	Treated with EDTA

STORAGE AND SHIPPING:

1. Sample Storage

All samples should be stored in a frozen environment (-80°C).

Avoid freeze/thaw cycles as this will negatively affect sample integrity.

Ensure that all samples are labeled with patient ID, type of sample and date and time of draw.

Each ID number should be associated with either a paper card orelectronic manifest including the following information:

2. Sample Shipping

All samples should be shipped in a frozen environment (-80°C).

Avoid freeze/thaw cycles, as this will damage the sample integrity.

Ensure that all samples are labeled with patient ID, type of sample and date and time of draw.

Shipments should include either card information or electronic manifestassociated with patient ID numbers.

First shipment should be sent once 100 serum and 100 plasma samples have been collected.

Second shipment should be sent at completion of study.

Shipping Address

Notify study collaborators of the shipment within 24 hours of sending the package. In this email, include the following:

Collection Site

Date of shipment

Tracking number

Number of samples sent

Electronic manifest summarizing the samples included in the shipment

Please name the file: YYYYMMDD Country_Shipment Manifest

Supplementary Material 2

Detailed description of the MS analytic pipeline for data acquisition, QA/QC, annotation, and structure identification

Deep metabolomic profiling pipeline and data analysis processes

Serum samples were retrieved from -80°C freezers and thawed on ice. A mixture of 240 μL extraction buffer (stored at -20°C) consisting of methanol, acetonitrile and ddH_2O (5:3:2 v/v) was added to 10 μL of each sample. Samples were vortexed at 4°C and then centrifuged at 12,000 g for 30 min at the same temperature. Supernatant (170 μL) from each sample was transferred to a vial and stored at -20°C before injection.

MS acquisition

Flow-injection analysis (FIA) was performed with QE-Plus mass spectrometer (Thermo Fisher, Shanghai, China). Samples were injected at 10 μL each for 3 min with mobile phase (flow rate at 50 μL/min) consisting of 0.1 % formic acid in 5% acetonitrile. The injected samples were sprayed at 4 kV and 325°C and scanned with a m/z range of 60-900 at a resolution of 140,000. Ionization modes were set to both positive and negative in turn to collect complete metabolic profiles for each sample. Meanwhile, these samples were also analyzed with liquid chromatography-tandem mass spectrometry (LC-MS/MS)

in data-dependent acquisition (DDA) mode according to a protocol previously published [1].

MS QA/QC

Several types of controls were analyzed in concert with the experimental samples: testing matrix sample, a pool generated by taking a small volume of each experimental sample; QC matrix sample, a pool of well-characterized human sera (Sigma-Aldrich, S1-M, human serum, normal) served as a technical replicate throughout the data sets; extracted water samples served as process blanks. Instrument variability was determined by calculating the median relative standard deviation (RSD) for the standards that were added to each sample prior to injection into the mass spectrometers. Bland–Altman plots [2] were used in analyzing the agreement between two different runs for the quality control. Overall process variability was determined by calculating the median RSD for all endogenous metabolites present in 100% of the pooled QC matrix samples. Experimental samples were randomized across the platform runs. To mitigate the potential blood processing and batch variance, QC matrix samples were spaced evenly (every 10 testing sera) among the injections to ensure intra- and inter-batch consistency (Supplementary Table 4) from metabolomics analytics. All the data analyses in this study were performed using R (version 4.0.3) programming language

and R software packages. Univariate statistical analysis was performed with R Stats (version 4.0.3, statistics analytics) package.

MS data processing

Raw MS files were converted and centroided with ProteoWizard and saved to mzXML files [3]. Injection zones and m/z bands were detected with proFIA (version 1.15.0, mass spectrometry data preprocessing analytics) package [4]. Detected bands were then aligned to derive data matrix across samples in the same batch with MzClust method from xcms (version 3.12.0, high resolution mass spectrometric spectra analytics) package [5]. Robust locally estimated scatterplot smoothing (LOESS) model was applied to each set of aligned bands to remove intra-batch drifting along injection process [6]. Inter-batch difference was removed by normalizing to the median of normal baseline per set of aligned bands (namely metabolic features). Metabolic features with more than 30% missing values were removed. The filtered data matrix was $\log_2$ transformed, centered, and scaled with the means and standard deviations (SDs) of the normal population in each metabolic feature. Finally, missing values were imputed with k-nearest neighbor (kNN) algorithm from impute (version 1.64.0, KNN imputation analytics) package.

Mass spectrometric feature annotation

Healthy general population subjects were split randomly into either training (75%) or testing (25%) sets. During the downstream elastic net analysis of the

age associated pathways, different univariate |r| thresholds (0.3, 0.4, 0.5, 0.6) were used for the selection of LCMS features correlating to the chronological age. Supplementary Fig. 1 summarized the resulting pathway based elastic net model performance as a function of different |r| thresholds.

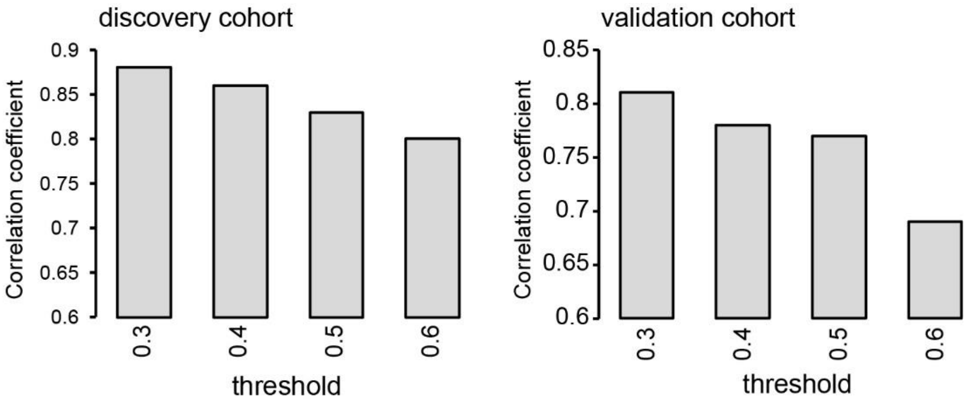

Supplementary Figure 1. Performances of the pathway based multivariate modeling as a function of the univariate |r| thresholds. Prediction performance is evaluated as a correlation coefficient between the predicted metabolic age and the chronological age. Training (75%discovery cohort) and testing (25%, validation cohort) sets were constructed from the healthy general population.

LCMS metabolic features in the training set were first correlated with chronological age and those with absolute correlation coefficients $|r| \geq 0.3$ were preserved. As defined by the Metabolomics Standards Initiative[7], there

are four levels of metabolite identification confidence: confidently identified compounds (level 1), putatively annotated compounds (level 2), and putatively annotated compound classes (level 3) and unknown compounds (level 4). The preserved features were annotated (level 2 or 3) with 5 ppm tolerance with xMSannotator[8] via KEGG compound data base[9].

Structural identification and metabolite biomarker discovery

Definitive (level 1) identification requires comparing the two or more orthogonal properties such as retention time, m/z, and fragmentation mass spectrum for the metabolite of interest to the same properties of an authentic chemical standard observed under identical analytical conditions. Metabolite biomarker identification was performed as a level 1 identification with chemical standards according to MSI [10]. With tandem mass spectrometry (MS/MS, Thermo Q Exactive plus, Thermo Fisher) data of serum samples and manual review confirmation, the generated MS1/MS2 pairs were searched in the public databases: HMDB[11] (http://www.hmdb.ca/), MoNA (http://mona.fiehnlab.ucdavis.edu/), MassBank (http://www.massbank.jp/), METLIN[12] (https://metlin.scripps.edu), and NIST (https://www.nist.gov/). The metabolites of interest were procured and subjected to a level 1 identification comparing the retention time, MS1 and MS2 patterns with the biomarker candidates, using the same LCMS/MS protocol with the sample analysis.

Metabolite biomarker candidates were selected from the metabolites that met all the following criteria: (a) the metabolite appeared in the contributing pathways with importance score > 0; (b) the metabolite's corresponding metabolic feature met $|r| \geq 0.3$; (c) the metabolite was validated with structural identification.

Among the changing metabolites between the healthy general population and CRC patient samples, the major metabolic modules were "citrate cycle", "tyrosine metabolism" and "valine, leucine degradation", where similar changes across all stages were observed (Supplementary Fig. 2 in supplementary material 3). The citrate cycle serves as the central hub of energy metabolism and is well known for its dysregulation in oncogenesis.[13] Two metabolic modules were found to be stage-specific: "pentose and glucuronate conversion" and "ascorbate and aldarate metabolism". Furthermore, we also identified a wide variety of metabolites scattering in many other metabolic modules, including "steroid hormone biosynthesis" and "oxidative phosphorylation". Many oncogenes and tumor suppressors regulate in a bi-directional manner the expression of fuel transporters and activity of cycle-related enzymes in cancer cells.[14] These enzymes include aconitase (also known as aconitate hydratase, AH), isocitrate dehydrogenase (IDH), fumarase (FH), succinate dehydrogenase (SDH) and α-ketoglutarate

dehydrogenase complex (KGDHC).[15-17] Our study validated the dysregulation of IDH activity by changes that occurred in isocitrate and oxalosuccinate levels (compounds at both ends of reaction) at the same time, while proposing other possibly changed reactions among oxaloacetate, citrate, and isocitrate, such as citrate synthase (oxaloacetate to citrate) and citrate hydro-lyase (citrate to isocitrate). There were two metabolites with significant changes in tyrosine metabolism. Homovanillate has been reported as a biomarker for breast cancer and colorectal cancer in urine [18, 19]. Regulated by tyrosine through levodopa (L-DOPA), homovanillate is the major product of monoamine oxidase and catechol-O-methyltransferase on dopamine. 3-(4-hydroxyphenyl)lactate has also been reported as a biomarker for colorectal cancer in both plasma and urine,[20] regulated by 3-(4-hydroxyphenyl) pyruvate. Although these two metabolites have been studied in CRC detection, our study unveiled their correlation with the aging process, suggesting them as possible evidence of oncogenic alterations on aging. On the other hand, sugar alcohols including xylitol, lyxitol, and ribitol are age-specific in microbiota of mice, by changing host inflammatory and metabolic signaling such as insulin sensitivity.[21] Our study shed light on the dysregulation of these metabolites as possible age-related biomarker candidates in human colorectal cancer in its early stages. In general, our study constructed a high-resolution panoramic view of metabolic changes in

CRC samples against the metabolic aging clock trained from a healthy general population with nine age-related metabolites, complementing and revealing the underlying biology of both aging and oncogenic pathophysiology.

Our study revealed progressive alteration of metabolic profiles as a function of different CRC stages (Supplementary Fig. 2 in supplementary material 3). During APL and stage I, metabolites in "pentose and glucuronate conversion" and "ascorbate and aldarate metabolism" modules were dysregulated in correlation with chronological age, resulting in a hypo-aging trend. Bioinformatics analysis combined with experimental verification in colorectal cancer found differentially expressed hub genes significantly enriched in the "pentose and glucuronate conversion" pathway[22]. Changes in metabolic driver genes were observed at the initial stages of oncogenesis,[23] and glucose transport and anaerobic metabolism have been studied in cancers (including CRC) for over a decade [24]. Both "pentose and glucuronate conversion" and "starch as sucrose metabolism" are downstream modules of glycolysis on metabolic pathways (hsa01100). This is in line with our observation that metabolites in the "pentose and glucuronate conversion" module are dysregulated accordingly during the early stages of CRC.

Another meta-analysis study of colorectal cancer discovered that differentially expressed metabolites enriched in the ascorbate and aldarate metabolism in and all the other four modules above[25, 26]. Our study showed the correlation of metabolites in "pentose and glucuronate conversion" and "ascorbate and aldarate metabolism" as major contributors in the metabolic aging clock. Although our study revealed the dysregulated ascorbate and aldarate metabolism in the early stages of CRC, including APL and stage I CRC, the temporal correlations of metabolites in these two modules were to certain extent re-established during stage II and III CRC. Possibly, the cancer-associated hypoxia rewires the metabolism and re-activates glycolysis during CRC development [27].

Z statistics test analysis

Cohort sex-composition impact on the metabolomic clock performance was performed as follows. Let $\hat{\rho}_M$ and $\hat{\rho}_F$ be the observed correlation coefficients of the metabolomic clock in males and female testing cohorts, respectively. The test Z statistic in comparing them is given by

$$Z = \frac{\left[\frac{1}{2}\log\left(\frac{1+\hat{\rho}_F}{1-\hat{\rho}_F}\right) - \frac{1}{2}\log\left(\frac{1+\hat{\rho}_M}{1-\hat{\rho}_M}\right)\right]}{\sqrt{\frac{1}{n_F-3}+\frac{1}{n_M-3}}}$$

The *p*-value can be calculated as $P(|N(0,1)| > |Z|)$.

Similar Z statistics analysis was performed to evaluate the positive predictive value (PPV) differences when different approaches were used to detect APL and CRC.

REFERENCE

1. Liang L, Rasmussen M-LH, Piening B, Shen X, Chen S, Röst H, et al. Metabolic dynamics and prediction of gestational age and time to delivery in pregnant women. Cell. 2020; 181: 1680–92.
2. Giavarina D. Understanding Bland Altman analysis. Biochem Med (Zagreb). 2015; 25: 141-51.
3. Kessner D, Chambers M, Burke R, Agus D, Mallick P. ProteoWizard: open source software for rapid proteomics tools development. Bioinformatics. 2008; 24: 2534–6.
4. Delabrière A, Hohenester UM, Colsch B, Junot C, Fenaille F, Thévenot EA. *proFIA*: a data preprocessing workflow for flow injection analysis coupled to high-resolution mass spectrometry. Bioinformatics. 2017; 33: 3767–75.
5. Smith CA, Want EJ, O'Maille G, Abagyan R, Siuzdak G. XCMS: processing mass spectrometry data for metabolite profiling using nonlinear peak alignment, matching, and identification. Analytical Chemistry. 2006; 78: 779–87.
6. Dunn WB, Broadhurst D, Begley P, Zelena E, Francis-McIntyre S, Anderson N, et al. Procedures for large-scale metabolic profiling of serum and plasma using gas chromatography and liquid chromatography coupled to mass spectrometry. Nature Protocols. 2011; 6: 1060–83.
7. Sumner LW, Amberg A, Barrett D, Beale MH, Beger R, Daykin CA, et al. Proposed minimum reporting standards for chemical analysis Chemical Analysis Working Group (CAWG) Metabolomics Standards Initiative (MSI). Metabolomics. 2007; 3: 211-21.
8. Uppal K, Walker DI, Jones DP. xMSannotator: An R Package for Network-Based Annotation of High-Resolution Metabolomics Data. Anal Chem. 2017; 89: 1063-7.
9. Kanehisa M, Goto S. KEGG: kyoto encyclopedia of genes and genomes. Nucleic Acids Res. 2000; 28: 27-30.
10. Viant MR, Kurland IJ, Jones MR, Dunn WB. How close are we to complete annotation of metabolomes? Curr Opin Chem Biol. 2017; 36: 64-9.
11. Wishart DS, Tzur D, Knox C, Eisner R, Guo AC, Young N, et al. HMDB: the human metabolome database. Nucleic Acids Research. 2007; 35: D521–D6.
12. Smith CA, O'Maille G, Want EJ, Qin C, Trauger SA, Brandon TR, et al. METLIN: a metabolite mass spectral database. Therapeutic Drug Monitoring. 2005; 27: 747–51.
13. Anderson NM, Mucka P, Kern JG, Feng H. The emerging role and targetability of the TCA cycle in cancer metabolism. Protein & Cell. 2018; 9: 216–37.
14. Chen J-Q, Russo J. Dysregulation of glucose transport, glycolysis, TCA cycle and glutaminolysis by oncogenes and tumor suppressors in cancer cells. Biochimica et Biophysica Acta (BBA) - Reviews on Cancer. 2012; 1826: 370–84.
15. Eng C, Kiuru M, Fernandez MJ, Aaltonen LA. A role for mitochondrial enzymes in inherited neoplasia and beyond. Nature Reviews: Cancer. 2003; 3: 193–202.

16. Juang H-H. Modulation of mitochondrial aconitase on the bioenergy of human prostate carcinoma cells. Molecular Genetics and Metabolism. 2004; 81: 244–52.
17. Yan H, Parsons DW, Jin G, McLendon R, Rasheed BA, Yuan W, et al. IDH1 and IDH2 mutations in gliomas. New England Journal of Medicine. 2009; 360: 765–73.
18. Nam H, Chung BC, Kim Y, Lee K, Lee D. Combining tissue transcriptomics and urine metabolomics for breast cancer biomarker identification. Bioinformatics. 2009; 25: 3151–7.
19. Cheng Y, Xie G, Chen T, Qiu Y, Zou X, Zheng M, et al. Distinct urinary metabolic profile of human colorectal cancer. Journal of Proteome Research. 2012; 11: 1354–63.
20. Zarei I, Baxter BA, Oppel RC, Borresen EC, Brown RJ, Ryan EP. Plasma and urine metabolite profiles impacted by increased dietary navy bean intake in colorectal cancer survivors: a randomized-controlled trial. Cancer Prevention Research. 2021; 14: 497–508.
21. Sheng L, Jena PK, Hu Y, Wan Y-JY. Age-specific microbiota in altering host inflammatory and metabolic signaling as well as metabolome based on the sex. Hepatobiliary Surgery and Nutrition. 2021; 10: 31–48.
22. Zhou H, Yang Z, Yue J, Chen Y, Chen T, Mu T, et al. Identification of potential hub genes via bioinformatics analysis combined with experimental verification in colorectal cancer. Molecular Carcinogenesis. 2020; 59: 425–38.
23. Youn A, Simon R. Identifying cancer driver genes in tumor genome sequencing studies. Bioinformatics. 2011; 27: 175–81.
24. Airley RE, Mobasheri A. Hypoxic regulation of glucose transport, anaerobic metabolism and angiogenesis in cancer: novel pathways and targets for anticancer therapeutics. Chemotherapy. 2007; 53: 233–56.
25. Tian J, Xue W, Yin H, Zhang N, Zhou J, Long Z, et al. Differential metabolic alterations and biomarkers between gastric cancer and colorectal cancer: a systematic review and meta-analysis. OncoTargets and Therapy. 2020; 13: 6093–108.
26. Malila N, Virtanen M, Pietinen P, Virtamo J, Albanes D, Hartman AM, et al. A comparison of prospective and retrospective assessments of diet in a study of colorectal cancer. Nutrition and Cancer. 1998; 32: 146–53.
27. Muz B, de la Puente P, Azab F, Azab AK. The role of hypoxia in cancer progression, angiogenesis, metastasis, and resistance to therapy. Hypoxia. 2015; 3: 83–92.

Supplementary Material 3

We were among the first groups to propose a pathway-based computational methodology for chronological event prediction with global metabolomics [1]. Representing the biological grouping of individual metabolomic features to associate to targeted clinical outcomes, the pathway-based modeling analysis was found to have less variability and higher sensitivity than direct global metabolomic feature-based modeling.

Annotated mass spectrometric features were aggregated into relevant KEGG pathways, and the value of each pathway was calculated as the weighted sum of the normalized measurement values of metabolites on the pathway divided by the number of mapped metabolites. Specifically, univariate correlations r_i of each metabolic feature i were first calculated with chronological age. Next, feature directionalities (up/down) were calculated according to the positive/negative signs of the correlation coefficients. Then, the annotated metabolites were mapped to KEGG pathways k in each directionality as $P_{k,\text{ up/down}} = \{i | \forall \text{compound}_i \in \text{pathway}_k, r_i \gtrless 0\}$. Finally, pathway expression values p_{kj} for sample j were calculated with relevant metabolite serological abundance values f_{ij} as follows, where $\text{n}(A)$ stands for the number of elements in set A:

$$p_{kj,\text{ up/down}} = \frac{\sum_{i \in P_{k,\text{ up/down}}} (r_i f_{ij})}{\text{n}(P_{k,\text{ up/down}})}$$

The derived pathway matrix was used to perform the multivariate analysis by fitting an elastic net model using all pathways against age with tuned hyperparameters α and λ from cross validation (CV) on the training set to minimize the mean squared prediction error. The importance score of each pathway biomarker was measured by the absolute value of the estimated β coefficient of the normalized pathway level. The higher the importance score suggests a bigger role in predicting age. Caret (version 6.0-86, elastic net machine learning analytics) package [2] was used. Pathways with an importance score > 0 were defined as age predictor contributing pathways. Age predictions were made on both training (75%) and testing (25%) sets of the healthy general population.

To further unveil an overview of metabolic changes from the reference metabolic ageotype of healthy general population to CRC, aging associating metabolites were annotated in KEGG hsa01100 metabolic pathways. Pathview (version 1.30.1, KEGG metabolic pathway visualization analytics) package [3] in Cytoscape (KEGG metabolic pathway network visualization analytics) [4] was used with correlation coefficients of each sample group as color key (Supplementary Figure 2). Clusters of metabolites with significant correlations in the healthy general population were identified with KEGG modules and pattern changes in CRC samples were identified from these metabolite clusters.

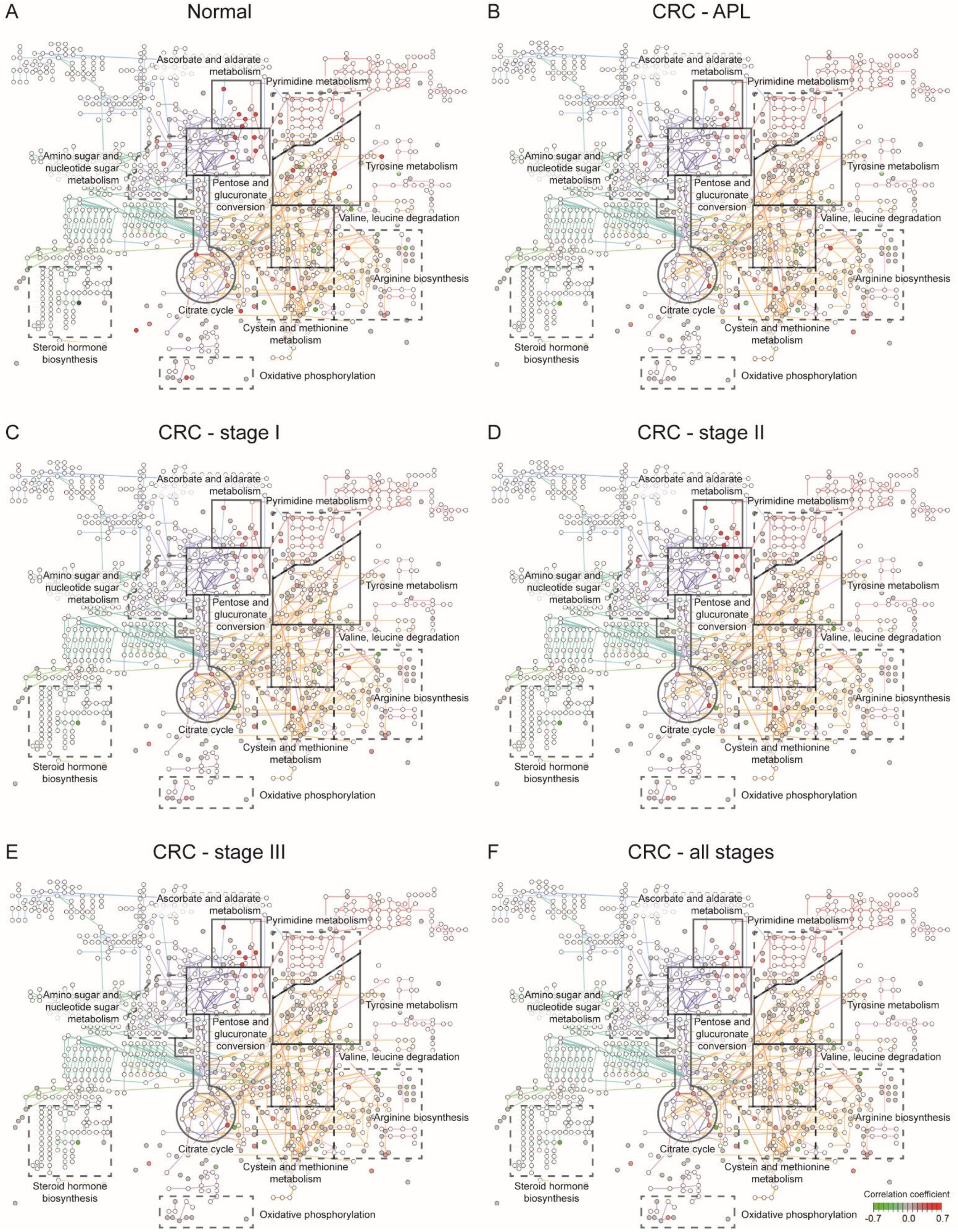
A
Normal
B
CRC - APL
C
CRC - stage I
D
CRC - stage II
E
CRC - stage III
F
CRC - all stages
Ascorbate and aldarate metabolism
Pyrimidine metabolism
Amino sugar and nucleotide sugar metabolism
Tyrosine metabolism
Pentose and glucuronate conversion
Valine, leucine degradation
Arginine biosynthesis
Citrate cycle
Cystein and methionine metabolism
Steroid hormone biosynthesis
Oxidative phosphorylation
Correlation coefficient
-0.7
0.0
0.7

Supplementary Figure. 2. Age-related metabolic alterations in colorectal cancer patients, relative to healthy individuals. With reference to **A.** healthy baseline, changes were observed in the "citrate cycle" and "tyrosine metabolism" for all CRC stages (**B-F**). There were also stage-specific alterations, including those in the "ascorbate and aldarate metabolism" for APL and stage I CRC (**B-C**), "pentose and glucuronate conversion" in all but stage I CRC (B, C, F) and "valine, leucine degradation" in all but stage Ill CRC **(B-D)**.

Among the changing metabolites between the healthy general population and CRC patient samples, the major metabolic modules were "citrate cycle", "tyrosine metabolism" and "valine, leucine degradation", where similar changes across all stages were observed (Supplementary Fig. 2). The citrate cycle serves as the central hub of energy metabolism and is well known for its dysregulation in oncogenesis [5]. Two metabolic modules were found to be stage-specific: "pentose and glucuronate conversion" and "ascorbate and aldarate metabolism". Furthermore, we also identified a wide variety of metabolites scattering in many other metabolic modules, including "steroid hormone biosynthesis" and "oxidative phosphorylation". Many oncogenes and tumor suppressors regulate in a bi-directional manner the expression of fuel transporters and activity of cycle-related enzymes in cancer cells [6]. These enzymes include aconitase (also known as aconitate hydratase, AH), isocitrate dehydrogenase (IDH), fumarase (FH), succinate dehydrogenase

(SDH) and α-ketoglutarate dehydrogenase complex (KGDHC) [7-9]. Our study validated the dysregulation of IDH activity by changes that occurred in isocitrate and oxalosuccinate levels (compounds at both ends of reaction) at the same time, while proposing other possibly changed reactions among oxaloacetate, citrate, and isocitrate, such as citrate synthase (oxaloacetate to citrate) and citrate hydro-lyase (citrate to isocitrate). There were two metabolites with significant changes in tyrosine metabolism. Homovanillate has been reported as a biomarker for breast cancer and colorectal cancer in urine [10, 11]. Regulated by tyrosine through levodopa (L-DOPA), homovanillate is the major product of monoamine oxidase and catechol-O-methyltransferase on dopamine. 3-(4-hydroxyphenyl)lactate has also been reported as a biomarker for colorectal cancer in both plasma and urine [12], regulated by 3-(4-hydroxyphenyl) pyruvate. Although these two metabolites have been studied in CRC detection, our study unveiled their correlation with the aging process, suggesting them as possible evidence of oncogenic alterations on aging. On the other hand, sugar alcohols including xylitol, lyxitol, and ribitol are age-specific in microbiota of mice, by changing host inflammatory and metabolic signaling such as insulin sensitivity [13]. Our study shed light on the dysregulation of these metabolites as possible age-related biomarker candidates in human colorectal cancer in its early stages. In general, our study constructed a high-resolution panoramic view of metabolic

changes in CRC samples against the metabolic aging clock trained from a healthy general population with nine age-related metabolites, complementing and revealing the underlying biology of both aging and oncogenic pathophysiology.

Our study revealed progressive alteration of metabolic profiles as a function of different CRC stages (Supplementary Fig. 2). During APL and stage I, metabolites in "pentose and glucuronate conversion" and "ascorbate and aldarate metabolism" modules were dysregulated in correlation with chronological age, resulting in a hypo-aging trend. Bioinformatics analysis combined with experimental verification in colorectal cancer found differentially expressed hub genes significantly enriched in the "pentose and glucuronate conversion" pathway [14]. Changes in metabolic driver genes were observed at the initial stages of oncogenesis [15], and glucose transport and anaerobic metabolism have been studied in cancers (including CRC) for over a decade [16]. Both "pentose and glucuronate conversion" and "starch as sucrose metabolism" are downstream modules of glycolysis on metabolic pathways (hsa01100). This is in line with our observation that metabolites in the "pentose and glucuronate conversion" module are dysregulated accordingly during the early stages of CRC.

Another meta-analysis study of colorectal cancer discovered that differentially expressed metabolites enriched in the ascorbate and aldarate metabolism in and all the other four modules above [17, 18]. Our study showed the correlation of metabolites in "pentose and glucuronate conversion" and "ascorbate and aldarate metabolism" as major contributors in the metabolic aging clock. Although our study revealed the dysregulated ascorbate and aldarate metabolism in the early stages of CRC, including APL and stage I CRC, the temporal correlations of metabolites in these two modules were to certain extent re-established during stage II and III CRC. Possibly, the cancer-associated hypoxia rewires the metabolism and re-activates glycolysis during CRC development [19].

REFERENCE

1. Sylvester KG, Hao S, You J, Zheng L, Tian L, Yao X, et al. Maternal metabolic profiling to assess fetal gestational age and predict preterm delivery: a two-centre retrospective cohort study in the US. BMJ Open. 2020; 10: e040647.
2. Kuhn M. Building Predictive Models in R Using the caret Package. Journal of Statistical Software. 2008; 28: 1 - 26.
3. Luo W, Brouwer C. Pathview: an R/Bioconductor package for pathway-based data integration and visualization. Bioinformatics. 2013; 29: 1830–1.
4. Shannon P, Markiel A, Ozier O, Baliga NS, Wang JT, Ramage D, et al. Cytoscape: a software environment for integrated models of biomolecular interaction networks. Genome Research. 2003; 13: 2498–504.
5. Anderson NM, Mucka P, Kern JG, Feng H. The emerging role and targetability of the TCA cycle in cancer metabolism. Protein & Cell. 2018; 9: 216–37.
6. Chen J-Q, Russo J. Dysregulation of glucose transport, glycolysis, TCA cycle and glutaminolysis by oncogenes and tumor suppressors in cancer cells. Biochimica et Biophysica Acta (BBA) - Reviews on Cancer. 2012; 1826: 370–84.
7. Eng C, Kiuru M, Fernandez MJ, Aaltonen LA. A role for mitochondrial enzymes in inherited neoplasia and beyond. Nature Reviews: Cancer. 2003; 3: 193–202.

8. Juang H-H. Modulation of mitochondrial aconitase on the bioenergy of human prostate carcinoma cells. Molecular Genetics and Metabolism. 2004; 81: 244–52.
9. Yan H, Parsons DW, Jin G, McLendon R, Rasheed BA, Yuan W, et al. IDH1 and IDH2 mutations in gliomas. New England Journal of Medicine. 2009; 360: 765–73.
10. Nam H, Chung BC, Kim Y, Lee K, Lee D. Combining tissue transcriptomics and urine metabolomics for breast cancer biomarker identification. Bioinformatics. 2009; 25: 3151–7.
11. Cheng Y, Xie G, Chen T, Qiu Y, Zou X, Zheng M, et al. Distinct urinary metabolic profile of human colorectal cancer. Journal of Proteome Research. 2012; 11: 1354–63.
12. Zarei I, Baxter BA, Oppel RC, Borresen EC, Brown RJ, Ryan EP. Plasma and urine metabolite profiles impacted by increased dietary navy bean intake in colorectal cancer survivors: a randomized-controlled trial. Cancer Prevention Research. 2021; 14: 497–508.
13. Sheng L, Jena PK, Hu Y, Wan Y-JY. Age-specific microbiota in altering host inflammatory and metabolic signaling as well as metabolome based on the sex. Hepatobiliary Surgery and Nutrition. 2021; 10: 31–48.
14. Zhou H, Yang Z, Yue J, Chen Y, Chen T, Mu T, et al. Identification of potential hub genes via bioinformatics analysis combined with experimental verification in colorectal cancer. Molecular Carcinogenesis. 2020; 59: 425–38.
15. Youn A, Simon R. Identifying cancer driver genes in tumor genome sequencing studies. Bioinformatics. 2011; 27: 175–81.
16. Airley RE, Mobasheri A. Hypoxic regulation of glucose transport, anaerobic metabolism and angiogenesis in cancer: novel pathways and targets for anticancer therapeutics. Chemotherapy. 2007; 53: 233–56.
17. Tian J, Xue W, Yin H, Zhang N, Zhou J, Long Z, et al. Differential metabolic alterations and biomarkers between gastric cancer and colorectal cancer: a systematic review and meta-analysis. OncoTargets and Therapy. 2020; 13: 6093–108.
18. Malila N, Virtanen M, Pietinen P, Virtamo J, Albanes D, Hartman AM, et al. A comparison of prospective and retrospective assessments of diet in a study of colorectal cancer. Nutrition and Cancer. 1998; 32: 146–53.
19. Muz B, de la Puente P, Azab F, Azab AK. The role of hypoxia in cancer progression, angiogenesis, metastasis, and resistance to therapy. Hypoxia. 2015; 3: 83–92.

Supplementary Material 4

Analytical pipeline and workflow.

Step 1: LCMS feature reduction

Out of the 1,603 LCMS features, 157 features (Pearson correlation, $|r| \geq 0.3$) were selected for downstream modeling analysis. To prepare for the elastic net analysis of age associated pathways, different univariate |r| thresholds (0.3, 0.4, 0.5, 0.6) were used for the selection of LCMS features correlating to the chronological age. Supplementary Figure 1 summarized the resulting pathway based elastic net model performance as a function of the univariate |r| thresholds.

Step 2: 157 LCMS feature mapping to metabolic pathways

The preserved 157 features were annotated (level 2 or 3) with 5 ppm tolerance with xMSannotator[1] via KEGG and HMDB compound data base[2]. Annotated mass spectrometric features were aggregated into relevant KEGG pathways (n=59), and the value of each pathway was calculated as the weighted sum of the normalized concentrations of metabolites on the pathway divided by the number of mapped metabolites.

Step 3: Elastic net method was used to construct a linear model to predict chronological age

With the elastic net analysis, 42/59 pathways were identified (beta coefficient not equal to 0) to contribute to the age prediction.

Step 4: Metabolic pathway based aging clock deviation analysis. To search for chronological deviations associated with pathologies of aging, a quantile linear regression model was fit with quantreg (version 5.75, quantile linear regression modeling analytics) package on predictions against chronological age from the normal population at 2.5%, 50% and 97.5%. The derived regression lines were overlaid with the predictions in both normal and CRC populations. Samples with predictions below the 2.5% quantile regression line was classified as “hypo-aging”, those with predictions above the 97.5% line as “hyper-aging”, and the rest (within 95% confidence interval) as normal aging, thus deriving three Δ age groups. Sample fractions in these Δ age groups were calculated in targeted categories of the interest.

Step 5: Structure identification

Supplementary Figure 3 (A). A histogram of all correlation (r) values obtained for the 157 annotated features. Black bars represent absolute correlation values ≥ 0.3, gray bars represent absolute correlation values < 0.3. The red dashed box represents the range of the nine features selected by the model.

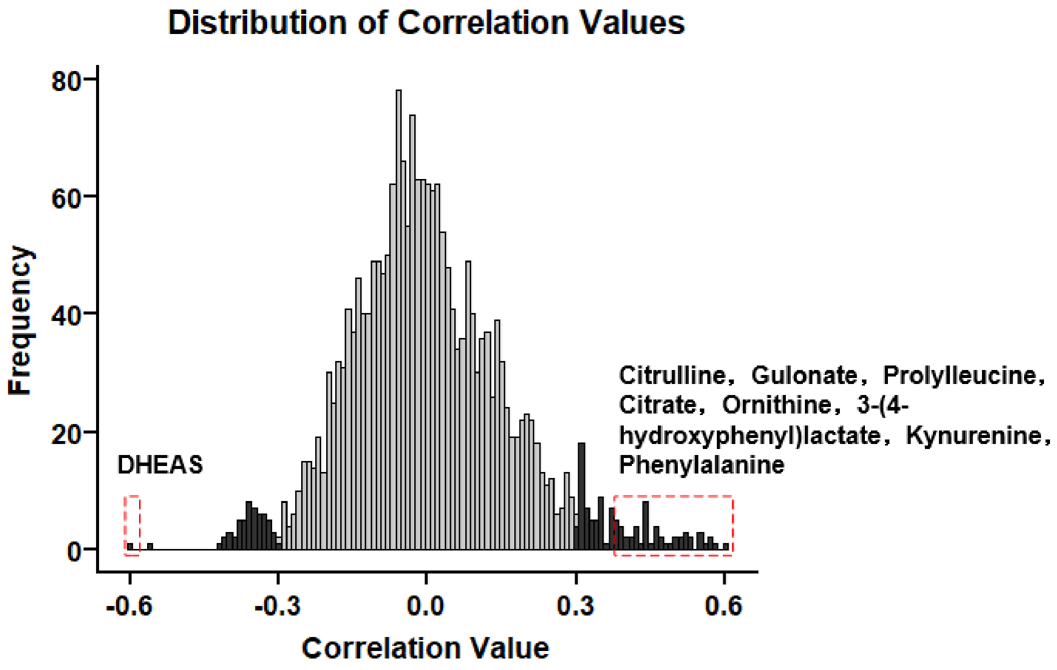

Supplementary Figure 3 (B). The match scores derived from the MS/MS spectra comparison of the metabolic clock nine metabolite markers. mzCloud: https://www.mzcloud.org/; MassBank: https://massbank.jp/Index.

Mode	Compund Name	mzCloud Score	MassBank Score	Adduct	m/z
Postitive	Phenylalanine	94.7		M+ACN+H	207.1101
				M+H	166.0855
	Prolylleucine	75.4		M+H	229.1543
	Citrulline		99.4	M+H	176.1023
	Kynurenine	86.3		M+H	209.0917
Negative	Ornithine	77.6		M-H	131.0825
	3-(4-Hydroxyphenyl)lactate	83.7		M-H	181.0505
	Citrate		89.3	M-H	191.0197
	Gulonate		62.1	M-H	195.0519
	DHEAS	89.4		M-H	367.1580

To identify the structures of LCMS features, we combined two pools of features:

1. Features mapped to HMDB but not KEGG: We selected features with absolute correlation values (r) greater than a threshold of 0.5.
2. Features mapped to 42 age-associated pathways: We included all mapped features from these pathways.

The combined pool of features contained 137 features, which we subjected to structure characterization and identification. We were able to determine the structures of 49 of these features.

Step 6: Construction of the nine-metabolite-based aging clock

Through an elastic net regularized regression ($\alpha = 0.125$, and $\lambda = 0.129$), a metabolic aging clock was trained with the metabolite biomarker candidates. The penalty parameters of the elastic net were chosen via cross validations to minimize the mean squared prediction error. The importance score of each metabolite biomarker was measured by the absolute value of the estimated b coefficient of the normalized metabolite level. The higher the importance score suggests a bigger role in predicting age. With positive importance scores, nine metabolites were identified to as the panel ("metabolic aging clock") to regress to the chronological age, deriving the metabolic age for each healthy or CRC subject. Regarding the metabolite-based metabolic clock, all the final 9 biomarkers' univariate |r| are > 0.4.

Step 7: The nine-metabolite-based aging clock deviation analysis.

Same algorithmic process will be used as Step 4.

With the elastic net analysis, 9/49 metabolites were identified (beta coefficient not equal to 0) to model the metabolic aging using a bootstrapped general population of Shanghai CDC balancing age and gender (Figure 3E/3F/3G). The resulted elastic net model was defined as the "metabolic aging clock". Comparative analysis of the enrolled individuals at Shanghai CDC and Fudan University cancer center revealed unique metabolic aging patterns associated with APL/CRC subjects (Figure 4A/4D/4G; Figure 5A/5C). A lower limit in our age prediction model is set at 30 years old.

To summarize, we only had one threshold (Pearson correlation, $|r| \geq 0.3$) to select LCMS features for downstream modeling analysis. Then we used two elastic net modeling processes to select pathways and structure-determined metabolic compounds for final linear modeling of chronological aging.

REFERENCES

1. Uppal K, Walker DI, Jones DP. xMSannotator: An R Package for Network-Based Annotation of High-Resolution Metabolomics Data. Anal Chem. 2017; 89: 1063-7.
2. Kanehisa M, Goto S. KEGG: kyoto encyclopedia of genes and genomes. Nucleic Acids Res. 2000; 28: 27-30.

Supplementary Material 5

Supplementary tables

Supplementary Table 1A. Demographics showing metabolic aging clock normal Δ aging subgroup distributions

	Normal	APL	CRC - stage I	CRC - stage II	CRC - stage III
**Total**	2860	290	377	277	264
**Age group, N (%)**					
**(30,40]**	15 (0.5)	14 (4.8)	7 (1.9)	2 (0.7)	15 (5.7)
**(40,50]**	469 (16.4)	47 (16.2)	62 (16.4)	43 (15.5)	37 (14)
**(50,60]**	1093 (38.2)	82 (28.3)	113 (30)	63 (22.7)	87 (33)
**(60,70]**	824 (28.8)	106 (36.6)	125 (33.2)	103 (37.2)	93 (35.2)
**(70,80]**	361 (12.6)	37 (12.8)	56 (14.9)	52 (18.8)	28 (10.6)
**(80,90]**	98 (3.4)	4 (1.4)	14 (3.7)	14 (5.1)	4 (1.5)
**Sex, N (%)**					
**Male**	1233 (43.1)	181 (62.4)	230 (61)	182 (65.7)	159 (60.2)
**Female**	1627 (56.9)	109 (37.6)	147 (39)	95 (34.3)	105 (39.8)

Supplementary Table 1B. Demographics showing metabolic aging clock hyper Δ aging subgroup distributions

	Normal	APL	CRC - stage I	CRC - stage II	CRC - stage III
**Total**	76	10	3	15	13
**Age group, N (%)**					
**(30,40]**	5 (6.6)	0 (0.0)	0 (0.0)	4 (26.7)	1 (7.7)
**(40,50]**	22 (28.9)	0 (0.0)	1 (33.3)	0 (0.0)	5 (38.5)
**(50,60]**	16 (21.1)	3 (30.0)	2 (66.7)	3 (20.0)	2 (15.4)
**(60,70]**	6 (7.9)	4 (40.0)	0 (0.0)	1 (6.7)	4 (30.8)
**(70,80]**	16 (21.1)	0 (0.0)	0 (0.0)	7 (46.7)	1 (7.7)
**(80,90]**	11 (14.5)	3 (30.0)	0 (0.0)	0 (0.0)	0 (0.0)
**Sex, N (%)**					
**Male**	35 (46.1)	8 (80)	2 (66.7)	12 (80)	11 (84.6)
**Female**	41 (53.9)	2 (20)	1 (33.3)	3 (20)	2 (15.4)

Supplementary Table 1C. Demographics showing metabolic aging clock hypo Δ aging subgroup distributions

	Normal	APL	CRC - stage I	CRC - stage II	CRC - stage III
**Total**	66	415	708	135	140
**Age group, N (%)**					
**(30,40]**	1 (1.5)	0 (0)	5 (0.7)	3 (2.2)	1 (0.7)
**(40,50]**	12 (18.2)	58 (14)	85 (12)	12 (8.9)	18 (12.9)
**(50,60]**	15 (22.7)	134 (32.3)	180 (25.4)	38 (28.1)	34 (24.3)
**(60,70]**	21 (31.8)	158 (38.1)	301 (42.5)	57 (42.2)	41 (29.3)
**(70,80]**	10 (15.2)	53 (12.8)	119 (16.8)	21 (15.6)	37 (26.4)
**(80,90]**	7 (10.6)	12 (2.9)	18 (2.5)	4 (3)	9 (6.4)
**Sex, N (%)**					
**Male**	44 (66.7)	221 (53.3)	391 (55.2)	78 (57.8)	64 (45.7)
**Female**	22 (33.3)	194 (46.7)	317 (44.8)	57 (42.2)	76 (54.3)

Supplementary Table 2A. Metabolic Δ aging subgroup membership, hyper/normal/hypo, determined by the nine-compound based metabolic aging clock

	**Normal**	**APL**	**CRC - stage I**	**CRC - stage II**	**CRC - stage III**
**Total**	3002	715	1088	427	417
**Δ age group, N (%)**					
**Hyper**	76 (2.5)	10 (1.4)	3 (0.3)	15 (3.5)	13 (3.1)
**Normal**	2860 (95.3)	290 (40.6)	377 (34.7)	277 (64.9)	264 (63.3)
**Hypo**	66 (2.2)	415 (58)	708 (65.1)	135 (31.6)	140 (33.6)

Supplementary Table 2B. Metabolic Δ aging subgroup memberships, hyper/normal/hypo, determined by the pathway based multivariate analysis.

	Normal	APL	CRC - stage I	CRC - stage II	CRC - stage III
**Total**	3002	715	1088	427	417
**Δ age group, N (%)**					
**Hyper**	102 (3.4)	36 (5)	46 (4.2)	63 (14.8)	56 (13.4)
**Normal**	2818 (93.9)	552 (77.2)	811 (74.5)	330 (77.3)	334 (80.1)
**Hypo**	82 (2.7)	127 (17.8)	231 (21.2)	34 (8)	27 (6.5)

Supplementary Table 2C. Classification performance after bootstrapping cohort samples to the true incidence rate in the general population.

Class	Model name	True positive	True negative	False positive	False negative	Sensitivity	Specificity
**CRC – APL**	**Metabolic clock panel**	13388	268873	7067	9419	58.70%	97.44%
**CRC - stage I~III**	**Metabolic clock panel**	1028	268873	7067	922	52.72%	97.44%
**CRC – APL**	**Multi-target panel**	9532	271539	4401	13268	41.81%	98.41%
**CRC - stage I~III**	**Multi-target panel**	1201	271539	4401	737	61.97%	98.41%

Supplementary Table 3A. Demographics showing sample distributions across age and sex in CRC stage I population

**Total**	412
**Age group, N (%)**	
**(30,40]**	6 (1.5)
**(40,50]**	56 (13.6)
**(50,60]**	102 (24.8)
**(60,70]**	154 (37.4)
**(70,80]**	76 (18.4)
**(80,90]**	18 (4.4)
**Sex, N (%)**	
**Male**	250 (60.7)
**Female**	162 (39.3)

Supplementary Table 3B. Demographics showing sample distributions across CRC mutations in CRC stage I population (N=412)

Mutant	KRAS	NRAS	BRAF
**Mutation, N (%)**	164 (39.8)	14 (3.4)	18 (4.4)

Supplementary Table 4. %CV of the biomarker metabolite abundance in QC matrix samples which were spaced every 10 testing sera

Biomarkers	Within-run %CV	Between-day %CV	Total %CV
Ornithine	6.4	4.7	7.9
3(4-Hydroxyphenyl)lactate	7.7	3.9	8.6
Citrate	7.7	3.9	8.6
Gulonate	7.8	3.6	8.6
DHEAS	8.5	4.5	9.6
Citrulline	6.5	4.8	8
Phenylalanine	4.5	6.4	7.8
Kynurenine	9	5.4	10.6
Prolylleucine	5.2	5.7	7.7

Supplementary Material 6

It is important to exclude potential technical bias in our study.

We have performed a follow up analysis in a subset of our cohort enrolled at Shanghai CDC, where identical blood collection was performed in CRC patients. We observed similar hypo-aging membership patterns in this subgroup of CRC patients, which supports the validity of our CRC results from Fudan University Shanghai Cancer Center. The following evidence was added in the discussion: "In a subset of our cohort enrolled at Shanghai CDC, identical blood collection was performed in healthy controls and CRC patients (n=55) who were identified as part of the CDC screening. Similar hypo-aging membership patterns were observed in this subgroup of CRC patients, (Figure. 5C-D), which supports the validity of our CRC results (Figure. 4A-B) from Fudan University Shanghai Cancer Center.

In addition, we developed 100 random models to assess whether the deviation of the CRC/APL cohort from the CI derived from CTRL is inherent to the age-related signature or due to other factors.

Supplementary Figure 4A shows the performance of 100 randomly built models to detect APL. All model PPVs are < 0.35, which is statistically lower (p-value < 0.05) than the original model (Table 2).

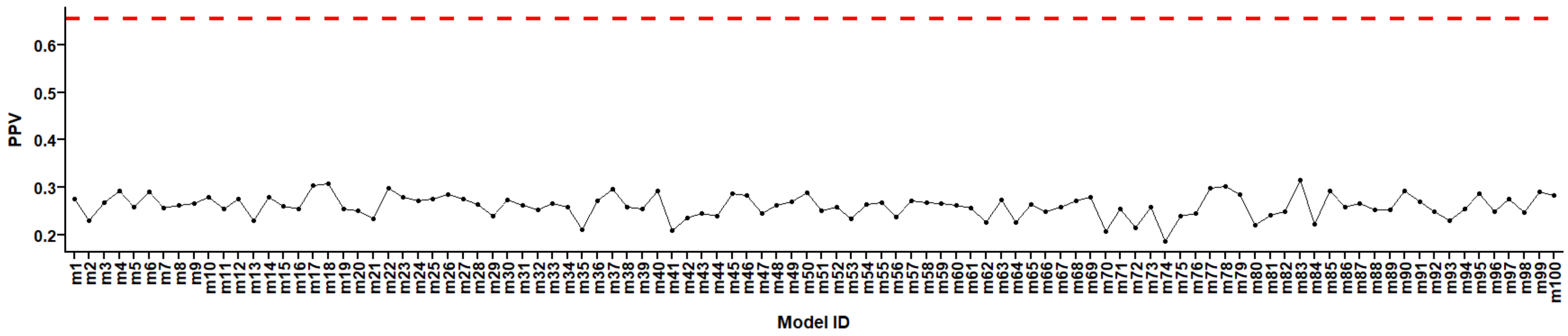

Supplementary Figure 4B shows the performance of 100 randomly built models to detect CRC. All model PPVs are < 0.35, which is statistically lower (p-value < 0.05) than the original model (Table 2).

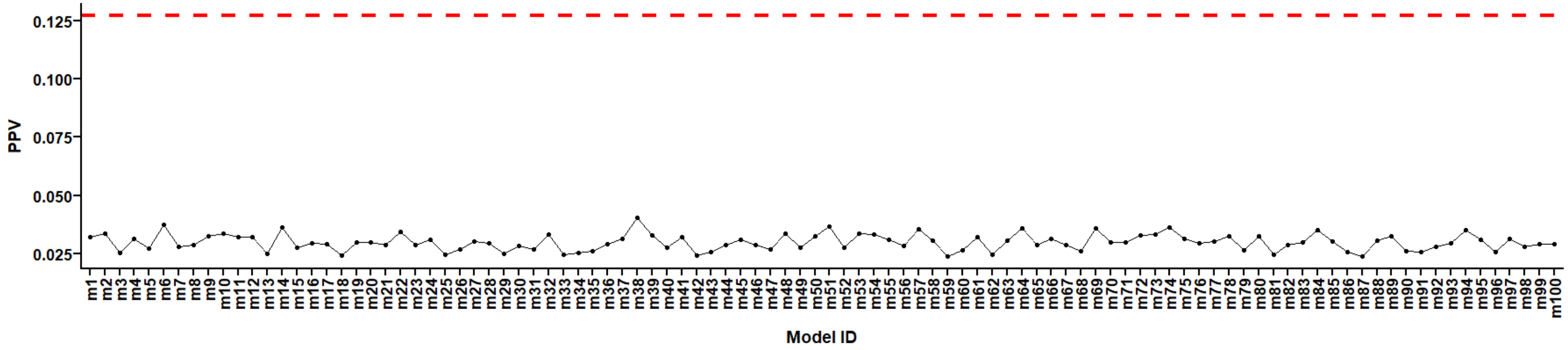

In summary, our computational analysis results suggest that our results are biologically meaningful and statistically significant. Our findings are unlikely to be due to technical bias.   This analysis supports our observations with the Shanghai CDC subset follow-up study,

Supplementary Material 7

Deep metabolomics profiling of healthy individuals revealed unique age-associated patterns in KEGG metabolic pathways. Each canonical pathway was divided into either a positive ("up") or negative ("down") age-correlated version based on the age correlation of its component compounds. An "up" pathway comprises significantly positive age-correlated pathway component compounds, while a "down" pathway comprises significantly negative age-correlated pathway component compounds. As detailed in Supplementary Material 3, the subject’s value of either "up" or "down" pathway was calculated as the weighted sum of the normalized measurement values of the corresponding “up” or “down” metabolites on the pathway divided by the number of mapped metabolites.

Supplementary Table 5 provides a detailed breakdown of these pathways and their associated mapped metabolite features, including pathway name, KEGG ID, total number of metabolites in the pathway, number of annotated metabolites, number of significant metabolites, names of significant metabolites within the pathways, and pathway rank in the all-combined men and women cohort, as well as in the men and women cohorts separately.
Supplementary Table 5. Aging associated KEGG metabolic pathways and their associated mapped metabolomic features.”

**Pathway name**	**Direction**	**Total number of metabolites in pathway**	**Number of metabolites annotated**	**Number of metabolites significant**	**Names of significant metabolites within pathways**	**Pathway rank in all**	**Pathway rank in men**	**Pathway rank in women**
map00140: Steroid hormone biosynthesis	down	99	5	1	Dehydroepiandrosterone sulfate;3beta-Hydroxyandrost-5-en-17-one 3-sulfate;DHEA sulfate; KEGG ID: C04555	1	1	2
map04976: Bile secretion	down	97	8	1	Dehydroepiandrosterone sulfate;3beta-Hydroxyandrost-5-en-17-one 3-sulfate;DHEA sulfate; KEGG ID: C04555	1	1	2

map02010: ABC transporters	up	137	45	12	D-Xylose;Wood sugar; KEGG ID: C00181<br>Hydroxyproline;L-Hydroxyproline;trans-4-Hydroxy-L-proline; KEGG ID: C01157<br>5-Aminolevulinate;5-Amino-4-oxopentanoate;5-Amino-4-oxovaleric acid; KEGG ID: C00430<br>Xylitol; KEGG ID: C00379<br>L-Arabinose;L-Arabinopyranose; KEGG ID: C00259<br>L-Ornithine;(S)-2,5-Diaminovaleric acid;(S)-2,5-Diaminopentanoic acid;(S)-2,5-Diaminopentanoate; KEGG ID: C00077<br>D-Methionine;D-2-Amino-4-(methylthio)butyric acid; KEGG ID: C00855<br>L-Cystine;L-Dicysteine;L-alpha-Diamino-beta-dithiolactic acid; KEGG ID: C00491<br>D-Sorbitol;D-Glucitol;L-Gulitol;Sorbitol; KEGG ID: C00794<br>Mannitol;D-Mannitol; KEGG ID: C00392<br>D-Ribose; KEGG ID: C00121<br>Uridine; KEGG ID: C00299	2	3	1
map00340: Histidine metabolism	down	47	17	1	3-(Imidazol-4-yl)-2-oxopropyl phosphate;Imidazole-acetol phosphate; KEGG ID: C01267	3	10	6
map00980: Metabolism of xenobiotics by cytochrome P450	down	121	11	1	N-Hydroxy-1-aminonaphthalene;1-Naphthylhydroxylamine; KEGG ID: C14789	4	4	14
map00740: Riboflavin metabolism	up	20	2	1	Ribitol;Adonitol; KEGG ID: C00474	5	12	4

map00360: Phenylalanine metabolism	up	60	26	8	Phenyllactate;(R)-Phenyllactate;(R)-3-(phenyl)lactate; KEGG ID: C05607<br>Benzoate;Benzoic acid;Benzenecarboxylic acid;Phenylformic acid;Dracylic acid; KEGG ID: C00180<br>cis-3-(3-Carboxyethenyl)-3,5-cyclohexadiene-1,2-diol;(2E)-3-(cis-5,6-Dihydroxycyclohexa-1,3-dien-1-yl)prop-2-enoate; KEGG ID: C12622<br>4-Hydroxy-2-oxopentanoate;4-Hydroxy-2-oxovalerate; KEGG ID: C03589<br>2-Hydroxy-2,4-pentadienoate;cis-2-Hydroxypenta-2,4-dienoate;Oxopent-4-enoate;2-Oxopent-4-enoate;2-Hydroxypenta-2,4-dienoate; KEGG ID: C00596<br>3-(2-Hydroxyphenyl)propanoate;2-Hydroxyphenylpropanoate;3-(2-Hydroxyphenyl)propionic acid;Melilotate;3-(2-Hydroxyphenyl)propionate; KEGG ID: C01198<br>3-(2,3-Dihydroxyphenyl)propanoate;2,3-Dihydroxyphenylpropanoate; KEGG ID: C04044<br>3-(3-Hydroxyphenyl)propanoic acid;Dihydro-3-coumaric acid;3-Hydroxyphenylpropanoate;3-(3-Hydroxyphenyl)propanoate; KEGG ID: C11457	6	2	NA
map05204: Chemical carcinogenesis	down	99	11	1	S-(1,2-Dichlorovinyl)-L-cysteine;DCVC; KEGG ID: C20317	6	9	8

map00020: Citrate cycle (TCA cycle)	up	20	7	4	Citrate;Citric acid;2-Hydroxy-1,2,3-propanetricarboxylic acid;2-Hydroxytricarballylic acid; KEGG ID: C00158<br>Oxaloacetate;Oxalacetic acid;Oxaloacetic acid;2-Oxobutanedioic acid;2-Oxosuccinic acid;keto-Oxaloacetate; KEGG ID: C00036<br>Oxalosuccinate;Oxalosuccinic acid; KEGG ID: C05379<br>Isocitrate;Isocitric acid;1-Hydroxytricarballylic acid;1-Hydroxypropane-1,2,3-tricarboxylic acid; KEGG ID: C00311	7	NA	5
map00240: Pyrimidine metabolism	up	65	19	3	Uridine; KEGG ID: C00299<br>Pseudouridine; KEGG ID: C02067<br>(R)-5,6-Dihydrothymine;(5R)-Dihydrothymine; KEGG ID: C21028	8	8	9
map00130: Ubiquinone and other terpenoid-quinone biosynthesis	up	92	11	1	(R)-3-(4-Hydroxyphenyl)lactate; KEGG ID: C03964	9	NA	7
map00760: Nicotinate and nicotinamide metabolism	up	55	20	3	2-Methyleneglutarate;alpha-Methylene glutarate; KEGG ID: C02930<br>2,3-Dimethylmaleate;Dimethylmaleic acid; KEGG ID: C00922<br>Methylitaconate;2-Methylene-3-methylsuccinate; KEGG ID: C02295	10	NA	21
map00260: Glycine, serine and threonine metabolism	down	50	22	2	Creatine;alpha-Methylguanidino acetic acid;Methylglycocyamine; KEGG ID: C00300<br>L-Tryptophan;Tryptophan;(S)-alpha-Amino-beta-(3-indolyl)-propionic acid; KEGG ID: C00078	11	NA	NA
map00480: Glutathione metabolism	up	38	6	2	L-Ornithine;(S)-2,5-Diaminovaleric acid;(S)-2,5-Diaminopentanoic acid;(S)-2,5-Diaminopentanoate; KEGG ID: C00077	12	17	15

map00040: Pentose and glucuronate interconversions	up	55	26	20	Xylitol; KEGG ID: C00379<br>L-Lyxonate;L-Lyxonic acid; KEGG ID: C05412<br>Ribitol;Adonitol; KEGG ID: C00474<br>L-Gulonate;L-Gulonic acid;Gulonate;Gulonic acid; KEGG ID: C00800<br>L-Arabitol;L-Arabinol;L-Arabinitol;L-Lyxitol; KEGG ID: C00532<br>D-Arabitol;D-Arabinitol;D-Arabinol;D-Lyxitol; KEGG ID: C01904<br>(4R,5S)-4,5,6-Trihydroxy-2,3-dioxohexanoate;2,3-Diketo-L-gulonate; KEGG ID: C04575<br>D-Ribulose;D-erythro-2-Pentulose;D-Arabinoketose;D-Arabinulose;D-Riboketose; KEGG ID: C00309<br>D-Xylulose;D-threo-Pentulose;D-Lyxulose; KEGG ID: C00310<br>L-Ribulose;L-erythro-Pentulose;L-Arabinoketose;L-Arabinulose;L-Riboketose; KEGG ID: C00508<br>L-Lyxose; KEGG ID: C01508<br>D-Xylose;Wood sugar; KEGG ID: C00181<br>D-Mannonate; KEGG ID: C00514<br>L-Arabinose;L-Arabinopyranose; KEGG ID: C00259<br>L-Galactonate;L-Galactonic acid; KEGG ID: C15930<br>D-Lyxose; KEGG ID: C00476<br>D-Xylonate; KEGG ID: C00502<br>L-Xylulose;L-threo-Pentulose;L-Lyxulose; KEGG ID: C00312<br>L-Xylonate;L-Xylonic acid; KEGG ID: C05411<br>D-Altronate; KEGG ID: C00817	13	13	23

map00270: Cysteine and methionine metabolism	down	63	19	1	L-Homocystine;Homocystine; KEGG ID: C01817	14	NA	27
map00480: Glutathione metabolism	down	38	6	1	Bis-gamma-glutamylcystine;Oxidized gamma-glutamylcysteine;Bis-gamma-L-glutamyl-L-cystine;Oxidized gamma-L-glutamyl-L-cysteine; KEGG ID: C03646	14	NA	11
map04261: Adrenergic signaling in cardiomyocytes	up	10	1	1	Isoproterenol;Isoprenaline; KEGG ID: C07056	14	23	12
map00250: Alanine, aspartate and glutamate metabolism	down	28	14	1	2-Oxosuccinamate;2-Oxosuccinamic acid;gamma-Aminooxaloacetate;Oxaloacetamide; KEGG ID: C02362	15	NA	18
map00760: Nicotinate and nicotinamide metabolism	down	55	20	1	Iminoaspartate;Iminoaspartic acid;Iminosuccinate; KEGG ID: C05840	15	NA	18
map04728: Dopaminergic synapse	up	12	4	2	3-Methoxytyramine; KEGG ID: C05587<br>Homovanillate;Homovanillic acid;3-Methoxy-4-hydroxyphenylacetate; KEGG ID: C05582	16	NA	NA
map00270: Cysteine and methionine metabolism	up	63	19	2	L-Cystine;L-Dicysteine;L-alpha-Diamino-beta-dithiolactic acid; KEGG ID: C00491<br>L-Methionine;Methionine;L-2-Amino-4methylthiobutyric acid; KEGG ID: C00073	17	NA	NA
map04974: Protein digestion and absorption	up	47	24	2	L-Methionine;Methionine;L-2-Amino-4methylthiobutyric acid; KEGG ID: C00073<br>L-Cystine;L-Dicysteine;L-alpha-Diamino-beta-dithiolactic acid; KEGG ID: C00491	17	11	25
map00260: Glycine, serine and threonine metabolism	up	50	22	2	5-Hydroxyectoine; KEGG ID: C16432<br>5-Aminolevulinate;5-Amino-4-oxopentanoate;5-Amino-4-oxovaleric acid; KEGG ID: C00430	18	NA	36

map00330: Arginine and proline metabolism	down	78	34	2	N-Methylhydantoin;N-Methylimidazolidine-2,4-dione; KEGG ID: C02565<br>Creatine;alpha-Methylguanidino acetic acid;Methylglycocyamine; KEGG ID: C00300	19	NA	NA
map00520: Amino sugar and nucleotide sugar metabolism	up	108	10	2	L-Arabinose;L-Arabinopyranose; KEGG ID: C00259<br>D-Xylose;Wood sugar; KEGG ID: C00181	20	10	NA
map00310: Lysine degradation	up	54	20	5	6-Amino-2-oxohexanoate;2-Oxo-6-aminocaproate; KEGG ID: C03239<br>L-2-Aminoadipate 6-semialdehyde;2-Aminoadipate 6-semialdehyde;L-Allysine;Allysine;(S)-2-Amino-6-oxohexanoate; KEGG ID: C04076<br>2-Amino-5-oxohexanoate; KEGG ID: C05825<br>(S)-5-Amino-3-oxohexanoic acid;(S)-5-Amino-3-oxohexanoate; KEGG ID: C03656<br>Glutarate;Glutaric acid;Pentanedioic acid;1,3-Propanedicarboxylic acid; KEGG ID: C00489	21	6	34
map00250: Alanine, aspartate and glutamate metabolism	up	28	14	2	Citrate;Citric acid;2-Hydroxy-1,2,3-propanetricarboxylic acid;2-Hydroxytricarballylic acid; KEGG ID: C00158<br>Oxaloacetate;Oxalacetic acid;Oxaloacetic acid;2-Oxobutanedioic acid;2-Oxosuccinic acid;keto-Oxaloacetate; KEGG ID: C00036	22	NA	NA
map00630: Glyoxylate and dicarboxylate metabolism	up	62	18	4	Isocitrate;Isocitric acid;1-Hydroxytricarballylic acid;1-Hydroxypropane-1,2,3-tricarboxylic acid; KEGG ID: C00311<br>trans-2,3-Epoxysuccinate; KEGG ID: C03548<br>Citrate;Citric acid;2-Hydroxy-1,2,3-propanetricarboxylic acid;2-Hydroxytricarballylic acid; KEGG ID: C00158<br>Oxaloacetate;Oxalacetic acid;Oxaloacetic acid;2-	22	NA	28

					Oxobutanedioic acid;2-Oxosuccinic acid;keto-Oxaloacetate; KEGG ID: C00036			
map04742: Taste transduction	up	32	9	1	Citrate;Citric acid;2-Hydroxy-1,2,3-propanetricarboxylic acid;2-Hydroxytricarballylic acid; KEGG ID: C00158	22	NA	38
map04922: Glucagon signaling pathway	up	26	8	3	Isocitrate;Isocitric acid;1-Hydroxytricarballylic acid;1-Hydroxypropane-1,2,3-tricarboxylic acid; KEGG ID: C00311<br>Citrate;Citric acid;2-Hydroxy-1,2,3-propanetricarboxylic acid;2-Hydroxytricarballylic acid; KEGG ID: C00158<br>Oxaloacetate;Oxalacetic acid;Oxaloacetic acid;2-Oxobutanedioic acid;2-Oxosuccinic acid;keto-Oxaloacetate; KEGG ID: C00036	22	NA	NA

map01210: 2-Oxocarboxylic acid metabolism	up	134	39	8	Oxalosuccinate;Oxalosuccinic acid; KEGG ID: C05379<br>L-Ornithine;(S)-2,5-Diaminovaleric acid;(S)-2,5-Diaminopentanoic acid;(S)-2,5-Diaminopentanoate; KEGG ID: C00077<br>Isocitrate;Isocitric acid;1-Hydroxytricarballylic acid;1-Hydroxypropane-1,2,3-tricarboxylic acid; KEGG ID: C00311<br>L-Methionine;Methionine;L-2-Amino-4methylthiobutyric acid; KEGG ID: C00073<br>Citrate;Citric acid;2-Hydroxy-1,2,3-propanetricarboxylic acid;2-Hydroxytricarballylic acid; KEGG ID: C00158<br>(S)-2-Acetolactate;(S)-2-Hydroxy-2-methyl-3-oxobutanoate;(2S)-2-Hydroxy-2-methyl-3-oxobutanoate; KEGG ID: C06010<br>3-Hydroxy-3-methyl-2-oxobutanoic acid;3-Hydroxy-3-methyl-2-oxobutanoate;2-Oxo-3-hydroxyisovalerate; KEGG ID: C04181<br>Oxaloacetate;Oxalacetic acid;Oxaloacetic acid;2-Oxobutanedioic acid;2-Oxosuccinic acid;keto-Oxaloacetate; KEGG ID: C00036<br>(R)-2,3-Dihydroxy-3-methylpentanoate;(R)-2,3-Dihydroxy-3-methylvalerate;(2R,3R)-2,3-Dihydroxy-3-methylpentanoate; KEGG ID: C06007	23	5	31
map00280: Valine, leucine and isoleucine degradation	down	42	14	2	(S)-3-Methyl-2-oxopentanoic acid;(S)-3-Methyl-2-oxopentanoate;(3S)-3-Methyl-2-oxopentanoic acid;(3S)-3-Methyl-2-oxopentanoate; KEGG ID: C00671<br>4-Methyl-2-oxopentanoate;2-Oxoisocaproate; KEGG ID: C00233	24	16	26

map00290: Valine, leucine and isoleucine biosynthesis	down	23	14	2	(S)-3-Methyl-2-oxopentanoic acid;(S)-3-Methyl-2-oxopentanoate;(3S)-3-Methyl-2-oxopentanoic acid;(3S)-3-Methyl-2-oxopentanoate; KEGG ID: C00671<br>4-Methyl-2-oxopentanoate;2-Oxoisocaproate; KEGG ID: C00233	24	NA	NA
map00472: D-Arginine and D-ornithine metabolism	up	11	7	5	L-Ornithine;(S)-2,5-Diaminovaleric acid;(S)-2,5-Diaminopentanoic acid;(S)-2,5-Diaminopentanoate; KEGG ID: C00077<br>2-Amino-4-oxopentanoic acid;2-Amino-4-oxopentanoate;(2R)-2-Amino-4-oxopentanoate; KEGG ID: C03341<br>5-Amino-2-oxopentanoic acid;5-Amino-2-oxopentanoate;2-Oxo-5-amino-pentanoate;2-Oxo-5-aminopentanoate;alpha-Keto-delta-aminopentanoate;2-Oxo-5-aminovalerate; KEGG ID: C01110<br>D-Ornithine; KEGG ID: C00515<br>(2R,4S)-2,4-Diaminopentanoate;D-threo-2,4-Diaminopentanoate;2,4-Diaminopentanoate; KEGG ID: C03943	25	NA	20
map00380: Tryptophan metabolism	up	83	12	4	5-Hydroxyindoleacetate; KEGG ID: C05635<br>L-Kynurenine;3-Anthraniloyl-L-alanine; KEGG ID: C00328<br>2-Oxoindole-3-acetate;2-Oxoindole-3-acetic acid;2-Indolinone-3-acetate;2-(2-Oxo-1,3-dihydroindol-3-yl)acetate; KEGG ID: C22202<br>Formyl-5-hydroxykynurenamine; KEGG ID: C05647	26	NA	NA
map00982: Drug metabolism - cytochrome P450	up	87	12	1	5-Phenyl-1,3-oxazinane-2,4-dione; KEGG ID: C16596	26	NA	19

map04726: Serotonergic synapse	up	42	3	1	5-Hydroxyindoleacetate; KEGG ID: C05635	26	NA	19
map00330: Arginine and proline metabolism	up	78	34	9	cis-4-Hydroxy-D-proline; KEGG ID: C03440<br>5-Amino-2-oxopentanoic acid;5-Amino-2-oxopentanoate;2-Oxo-5-amino-pentanoate;2-Oxo-5-aminopentanoate;alpha-Keto-delta-aminopentanoate;2-Oxo-5-aminovalerate; KEGG ID: C01110<br>L-Glutamate 5-semialdehyde;L-Glutamate gamma-semialdehyde; KEGG ID: C01165<br>trans-3-Hydroxy-L-proline;trans-L-3-Hydroxyproline; KEGG ID: C05147<br>L-Ornithine;(S)-2,5-Diaminovaleric acid;(S)-2,5-Diaminopentanoic acid;(S)-2,5-Diaminopentanoate; KEGG ID: C00077<br>Hydroxyproline;L-Hydroxyproline;trans-4-Hydroxy-L-proline; KEGG ID: C01157<br>4-Acetamidobutanoate;N4-Acetylaminobutanoate; KEGG ID: C02946<br>cis-3-Hydroxy-L-proline;cis-3-Hydroxyproline; KEGG ID: C19706<br>Creatinine;1-Methylglycocyamidine; KEGG ID: C00791	27	NA	29
map00071: Fatty acid degradation	up	50	4	1	Glutarate;Glutaric acid;Pentanedioic acid;1,3-Propanedicarboxylic acid; KEGG ID: C00489	28	19	NA
map00650: Butanoate metabolism	up	42	13	1	(S)-2-Acetolactate;(S)-2-Hydroxy-2-methyl-3-oxobutanoate;(2S)-2-Hydroxy-2-methyl-3-oxobutanoate; KEGG ID: C06010	28	19	NA
map00750: Vitamin B6 metabolism	up	28	4	1	2-(Hydroxymethyl)-4-oxobutanoate;alpha-Hydroxymethyl succinate semialdehyde;2-Hydroxymethyl succinate semialdehyde; KEGG ID: C04106	28	19	NA

Supplementary Material 8

Elastic net regularized regression (ElasticNet) requires the following data prerequisites:

- Linearity: The relationship between the independent and dependent variables should be linear.
- Independence: The independent variables should be independent of each other.
- Normality: The dependent variable should be normally distributed.
- Homoscedasticity: The variance of the dependent variable should be equal across all levels of the independent variables.

Supplementary Figure 5. Evidence to support the appropriateness to use of ElasticNet in this study.

A.. The validation for linearity has already been demonstrated in Figure 3C. All nine metabolic features used in the model exhibit good monotonicity.

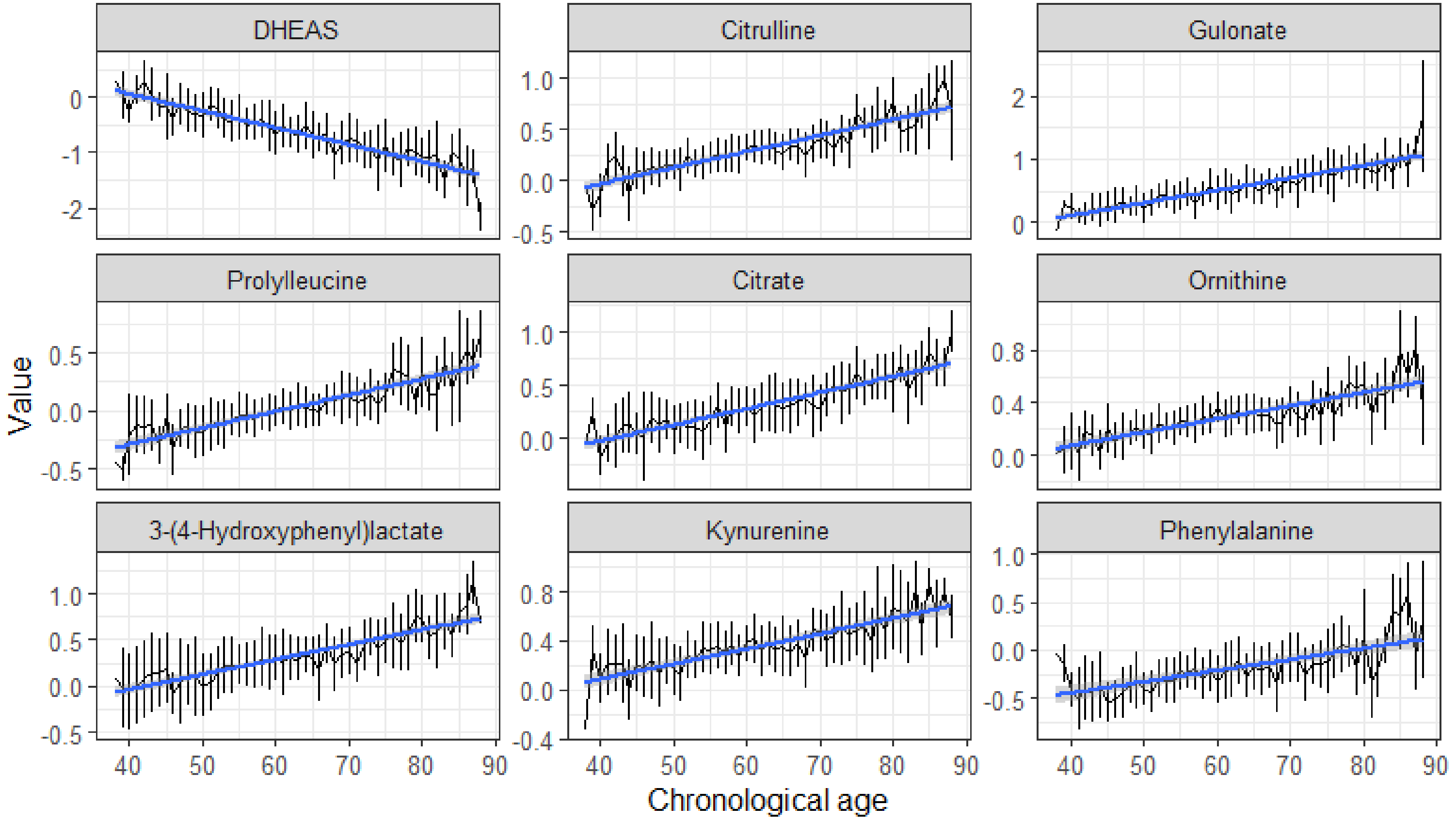

B. As shown in the heatmap below, the correlation coefficients between the features are all less than 0.5, indicating no significant inter-feature correlations and verifying the independence among the features. Furthermore, Elastic Net combines both L1 regularization (Lasso) and L2 regularization (Ridge), allowing it to simultaneously address variable selection (feature selection) and control model complexity. The presence of these two regularization terms enables Elastic Net to maintain model sparsity in the presence of multicollinearity. It selects the most important independent variables and pushes their coefficients towards zero while reducing the instability in coefficient estimation caused by multicollinearity.

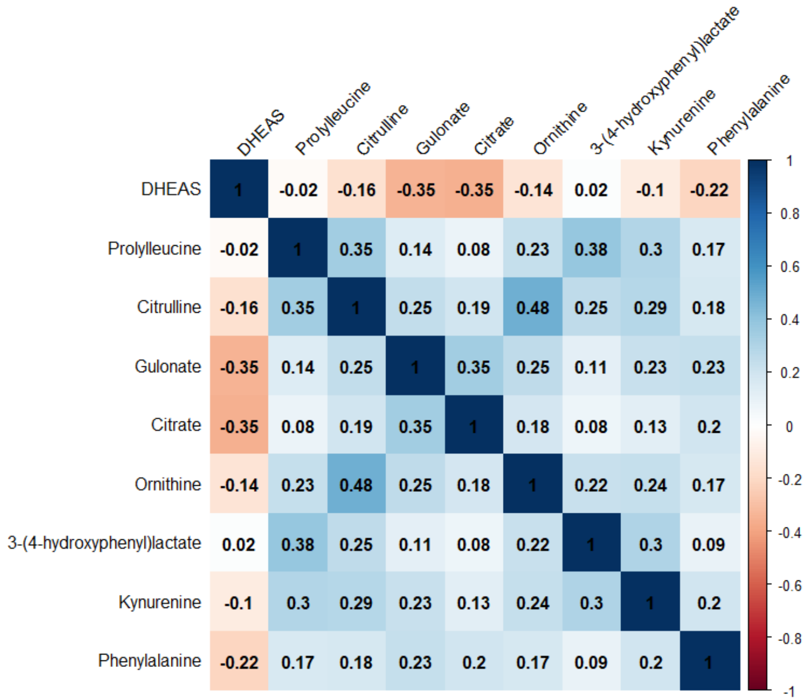

C. For the distribution of features, we used density plots for validation. Our plots show that each of the distributions of the nine features closely follows a normal distribution.

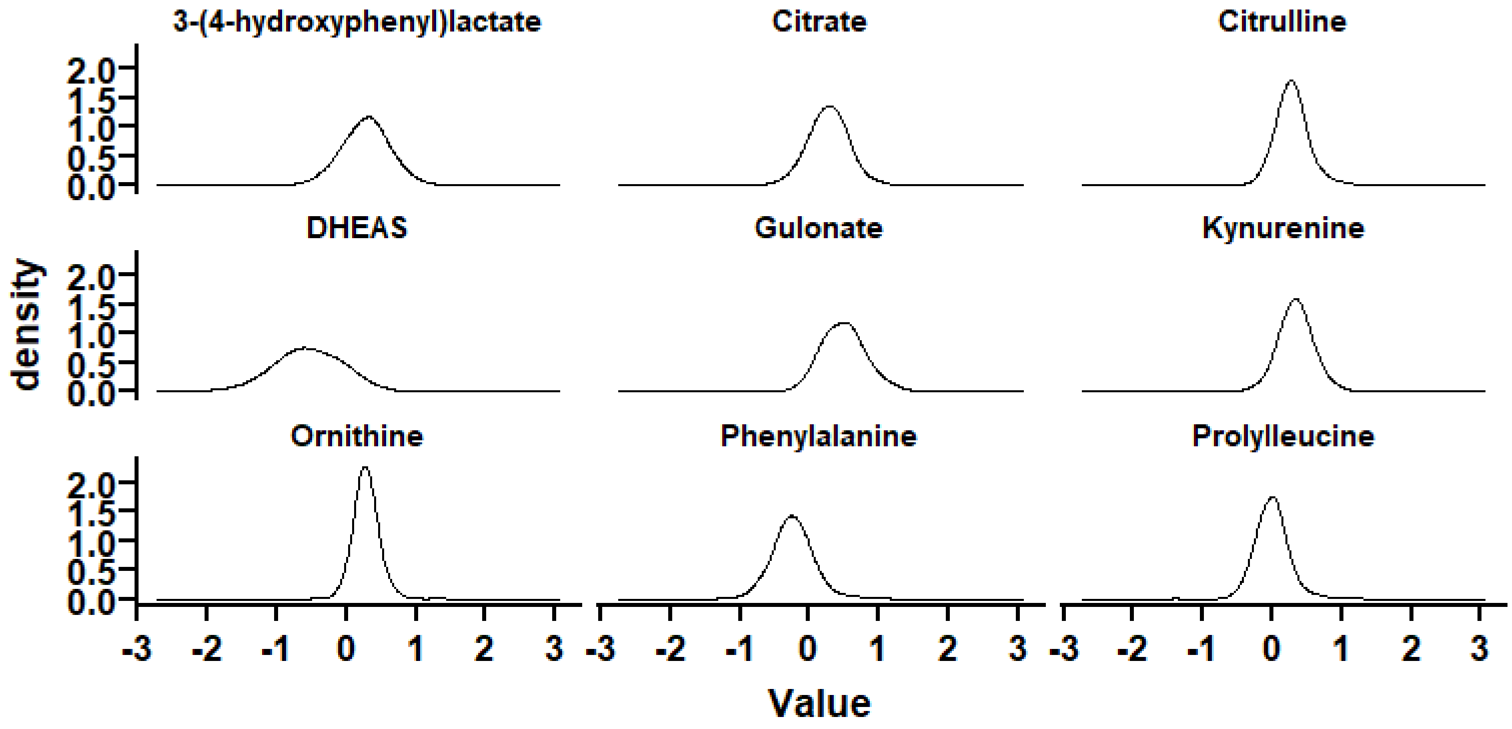

D. To address feature homoscedasticity, we applied a logarithmic transformation during data standardization to ensure the homoscedasticity of features. Additionally, we plotted residual graphs for each feature. From these graphs, it can be observed that the residuals for each feature exhibit random distribution, constant variance, and no apparent trends. Consequently, our dataset meets the requirement of equal variance.

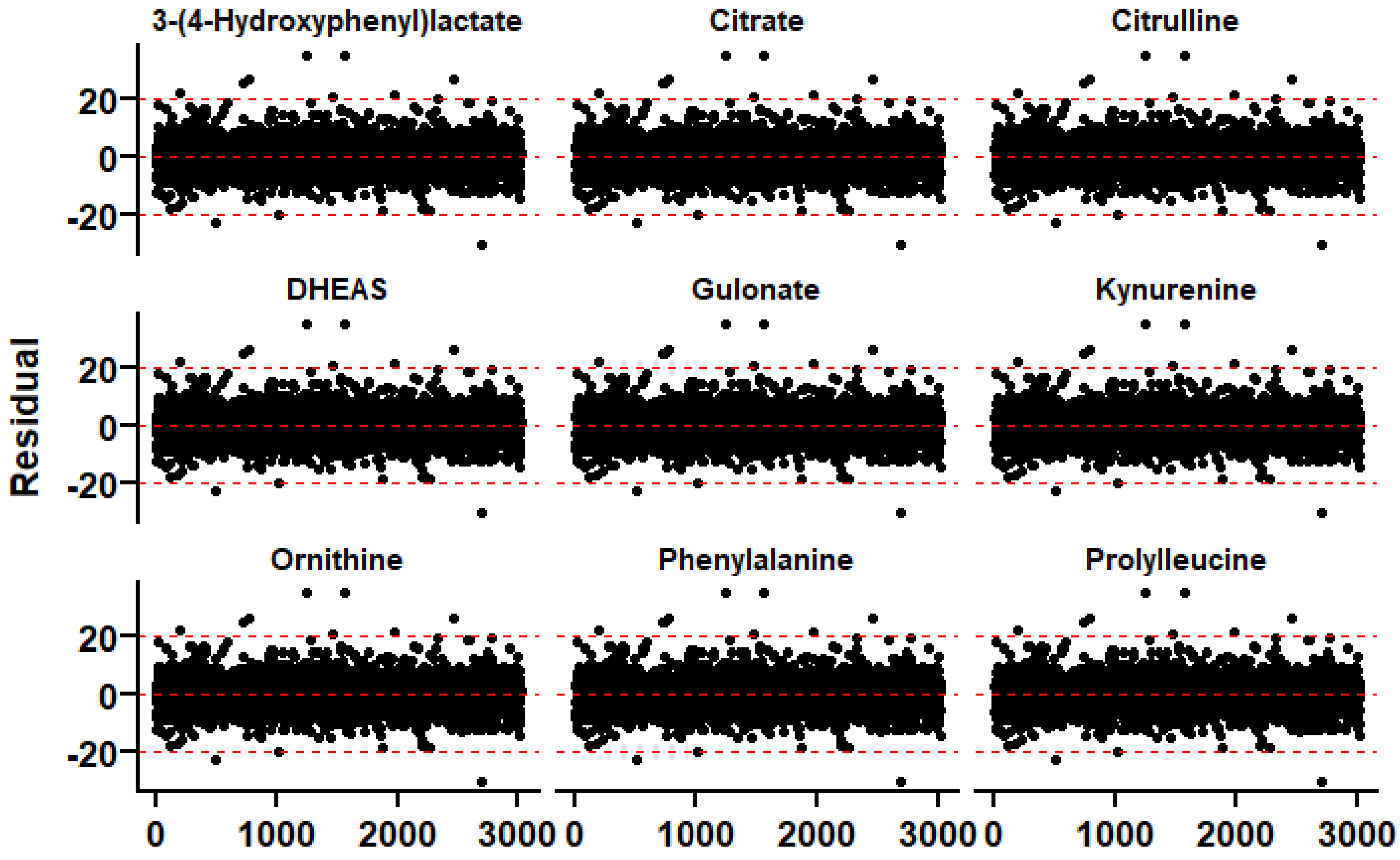

E. Regarding the issue of different gender distributions between the two datasets, we took into account the influence of gender on the model before modeling. Consequently, we independently established models for both the male and female populations and validated them on the cancer dataset. Therefore, we believe that the differing gender distributions will not affect the predictive results.
